# Driving a protective allele of the mosquito *FREP1* gene to combat malaria

**DOI:** 10.1038/s41586-025-09283-6

**Published:** 2025-07-23

**Authors:** Zhiqian Li, Yuemei Dong, Lang You, Rodrigo M. Corder, Jemariz Arzobal, Audrey Yeun, Lei Yang, John M. Marshall, George Dimopoulos, Ethan Bier

**Affiliations:** 1 Department of Cell and Developmental Biology, University of California, San Diego, La Jolla, CA USA; 2 Tata Institute for Genetics and Society, University of California, San Diego, La Jolla, CA USA; 3 Department of Molecular Microbiology and Immunology, Johns Hopkins Malaria Research Institute, Johns Hopkins University, Bloomberg School of Public Health, Baltimore, MD USA; 4 Department of Parasitology, Institute of Biomedical Sciences, University of São Paulo, São Paulo, Brazil; 5 Divisions of Biostatistics and Epidemiology, School of Public Health, University of California, Berkeley, CA USA; 6 Innovative Genomics Institute, Berkeley, CA USA

**Keywords:** Infectious-disease diagnostics, Population genetics

## Abstract

Malaria remains a substantial global health challenge, causing approximately half a million deaths each year^1^. The mosquito fibrinogen-related protein 1 (FREP1) is required for malaria parasites to infect the midgut epithelium^2^. The naturally occurring *FREP1*^*Q*^ allele has been reported to prevent parasite infection, while supporting essential physiological functions in the mosquito^3^. Here we generate congenic strains of *Anopheles stephensi*, edited to carry either the parasite-susceptible *FREP1*^*L224*^ or the putative-refractory *FREP1*^*Q224*^ alleles. The *FREP1*^*Q224*^ allele confers robust resistance to infection by both human and rodent malaria parasites, with negligible fitness costs. The protective *FREP1*^*Q224*^ allele can be efficiently driven into *FREP1*^*L224*^ mosquito populations using a novel linked allelic-drive system that selectively replaces the L224 codon with the parasite-refractory Q224 allele, thereby rendering populations refractory to parasite infection. This antimalaria drive system provides a novel genetic approach to aid in malaria elimination efforts.

## Abstract (web summary)

Coupling of a *FREP1* variant to a gene-drive system allows the spread of a host malaria resistance allele through the mosquito population.

## Main

Malaria remains one of the world’s most devastating diseases, claiming about 600,000 lives worldwide in 2023^1^. A decline in malaria deaths by approximately 50% during the past decade has been achieved primarily by the widespread use of insecticide-treated bed nets, indoor residual spraying of insecticides and antimalaria drugs^4^. However, these gains have been steadily eroding owing to the increasing prevalence of insecticide resistance in malaria mosquito vectors and the emergence of drug-resistant parasites^1,5^. An alternative and complementary approach is to develop genetically engineered mosquitoes that either suppress mosquito populations or modify them so that they can no longer sustain parasite transmission^5–11^. With regard to modification strategies, various exogenous and mosquito-encoded endogenous anti-*Plasmodium* effectors have been tested in mosquitoes. In addition, measures to disrupt the function of mosquito-expressed pathogen host factors have been shown to reduce parasite infection^12,13^. However, many challenges remain to be addressed with these approaches^5,6,8,14–20^. Concerns include: limited functionality of effectors due to imperfect synchronization between blood meal-inducible expression and parasite infection kinetics^8,21,22^; imposition of fitness costs (for example, modulating or disrupting expression of the endogenous target gene by genomic insertion of the transgenes)^23–25^; toxicity associated with high levels of effector expression; requirements for highly tissue-specific regulatory elements; and the potential for evolving mutations to impair effector functionality^6,23,26^. Furthermore, constitutive genetic inactivation of host factors often negatively affects mosquitoes by reducing viability and/or fertility, owing to a loss of biological functions beyond their roles in parasite infection^6,26^.

With the above considerations in mind, we envisioned an alternative streamlined strategy in which a genetic system preferentially biases the inheritance of a naturally occurring parasite-refractory allele of the host factor FREP1 that retains its essential physiological functions for the mosquito. FREP1 is a peritrophic matrix-associated factor that has been described as a promising host target for suppressing parasite infection owing to its important role in facilitating the traversal of malaria parasites across the midgut epithelium. This step has been described as a key bottleneck of the infection cycle of *Plasmodium* during the invertebrate stage, as only a few of the several thousands of ingested parasites successfully develop into oocysts between the midgut epithelium and its basal lamina^5,27,28^. A genome-wide association study identified a naturally occurring *FREP1* allelic variant in which L (leucine) is replaced by Q (glutamine) as a candidate polymorphism that confers resistance to *Plasmodium falciparum* transmission in *Anopheles gambiae* mosquitoes^3^. Subsequent antibody blocking and genetic studies have confirmed the essential role of FREP1 in parasite infection; however, the predicted protective role of the *FREP1*^*Q*^ allele has not yet been tested in a stable mosquito colony with a defined genotype, and correlative genetic evidence is currently absent for *FREP1* orthologues in other anopheline species^2,3,13,29,30^.

Here we generated congenic strains of *A.* *stephensi* differing only in a single amino acid residue that either carries the wild-type (WT) parasite-susceptible *FREP1*^*L224*^ allele or the putative parasite-refractory *FREP1*^*Q224*^ variant. We found that the *FREP1*^*Q224*^ allele retains essential functions for the mosquito but greatly reduces infection by both *P. falciparum* and *Plasmodium berghei* parasites at the pre-oocyst stage. We used a linked allelic-drive strategy to achieve efficient super-Mendelian transmission of this protective anti-*Plasmodium* allele, which should be readily transferable to other genetic loci and diverse *Anopheles* mosquito species, expanding the genetic toolbox for combating malaria.

## Generation of the *FREP1*^*Q*^ allelic variant

**Fig. 1:**
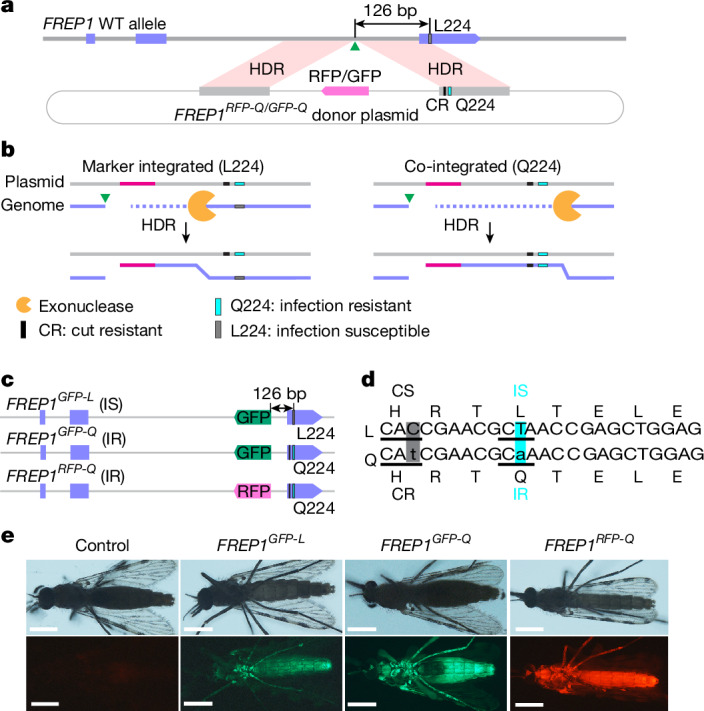
Generation of the *FREP1* allelic variants. **a**, Design of the donor plasmid. The bold grey line denotes genomic DNA at the *FREP1* locus; the grey boxes indicate homology arms; the circled light grey lines denote the plasmid backbone; the purple boxes indicate exons; and the green triangles indicate the gRNA cleavage site for gene cassette insertion. CR, cut resistant. **b**, Two possible editing outcomes: fluorescence marker insertion without (left) or with (right) the Q224 edit through homology-directed repair (HDR). The pink lines denote the RFP fluorescence marker. **c**, Established *FREP1* transgenic lines: *FREP1*^*GFP-L*^, *FREP1*^*GFP-Q*^ and *FREP1*^*RFP-Q*^. IR, infection resistant; IS, infection susceptible. **d**, Nucleotide and amino acid sequences of *FREP1* alleles carrying the L224 (top) or Q224 codon (bottom). The shaded boxes indicate altered nucleotides that either alter the codon for the same amino acid residue (the grey box denotes recoded cut susceptible (CS) > CR; C > t, H) or change an amino acid codon (the turquoise box denotes parasite IS > IR; T > a; L > Q). **e**, Fluorescent images of female transformants. Adult mosquitoes were imaged with a Zeiss Stemi 2000 fluorescence microscope, and images were processed with Fiji (OS version) and Photoshop (Photoshop CC v20.0.7). Scale bars, 0.5 mm. At least three individual mosquitoes for each line were used for imaging.

**Extended Data Fig. 1:**
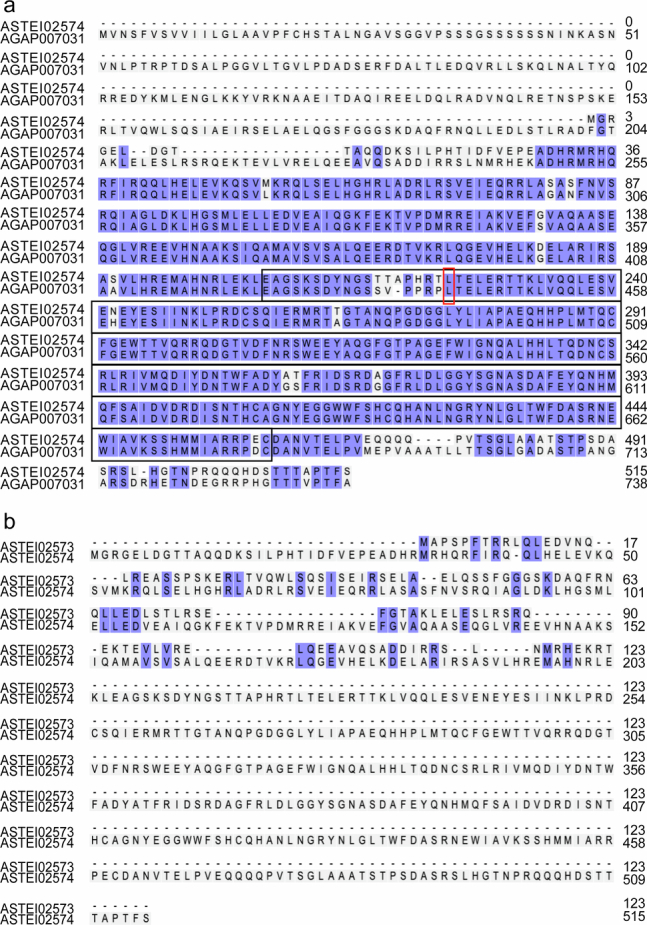
Multiple protein sequences alignment by CLUSTALW. **a**, Protein sequences alignment of FREP1 orthologs from *An. stephensi* and *An. gambiae*. **b**, Protein sequences alignment of two distantly related FREP1 paralogs from *An. stephensi*. Consensus amino acids are colored purple. The black rectangle in panel **a** indicates the conserved fibrinogen-like domain and the red rectangle denotes the Leucine residue for editing. Gene accession numbers are labeled on the left and the numbers on the right indicate the number of amino acid residues.

**Extended Data Fig. 2:**
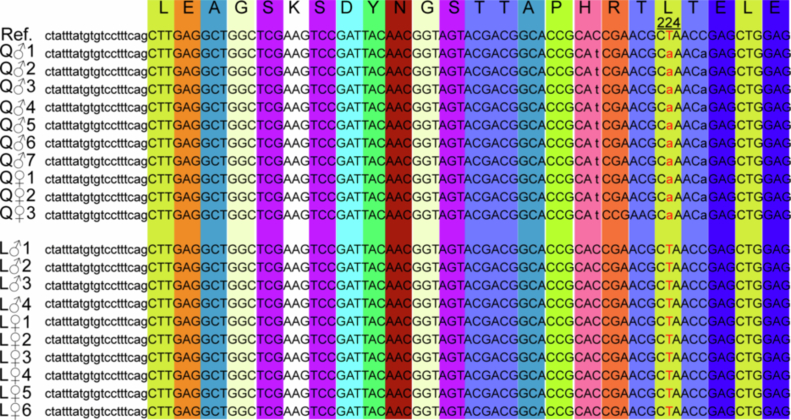
Genotyping of F_1_ transformants. F_1_ transformants were collected for genotyping after egg laying using primer sets amplifying sequences outside the two homology arms. The alignment displays partial *FREP1* locus sequences focused on the L224 amino acid. Amino acids are labeled with different colors and the T->A single nucleotide edit that recodes the L224 to Q224 is indicated in red. The reference WT sequence is shown on the top. Both female and male F_1_ transformants were used for genotyping.

We set out to rigorously test the parasite susceptibility of *FREP1* allelic variants in *A. stephensi*, the major Asian malaria mosquito vector by generating congenic strains that carry either the hypothesized parasite-refractory Q224 variant or the susceptible WT L224 allele (Extended Data Fig. 1). We recoded the L224 residue to Q224 (CTA to CAA) in the right homology arm flanking a selectable fluorescence marker cassette targeted for genomic insertion within the second intron of the *FREP1* gene using gRNA^Intron^ (cuts at 126 bp upstream of the T > A edit)^3^ (Fig. 1a). This design has several notable advantages in that it: (1) enables fluorescence marker-based screening of successful genomic integration events; (2) facilitates tracking of the allelic edit, which is tightly linked to a fluorescence marker; (3) provides congenic strains differing in only a single amino acid (L224 or Q224); and (4) causes minimal effects on endogenous *FREP1* gene activity (evidence presented below). We randomly selected and sequenced 20 F_1_ transgenic mosquitoes and found that co-integration of the Q224 edit with the fluorescence marker had occurred in half of the primary transformants (Fig. 1b). These results suggest that about 50% of the double-strand breaks (DSBs) generated by gRNA^Intron^ were resolved by homology-directed repair accompanied by a gene conversion tract comprising at least 126 bp, consistent with estimates of DSB resection lengths measured in mammalian cells, fruit flies and other species^31–35^ (Fig. 1b,c and Extended Data Fig. 2). We established three homozygous transgenic strains, two of which carried the Q224 allele but with differing fluorescence markers (*FREP1*^*GFP-Q*^ and *FREP1*^*RFP-Q*^), whereas the third strain carried the L224 allele with a GFP marker (*FREP1*^*GFP-L*^; Fig. 1c–e), which served as a WT control for subsequent experiments in this study.

## The *FREP1*^*Q*^ allele is fitness neutral

**Fig. 2:**
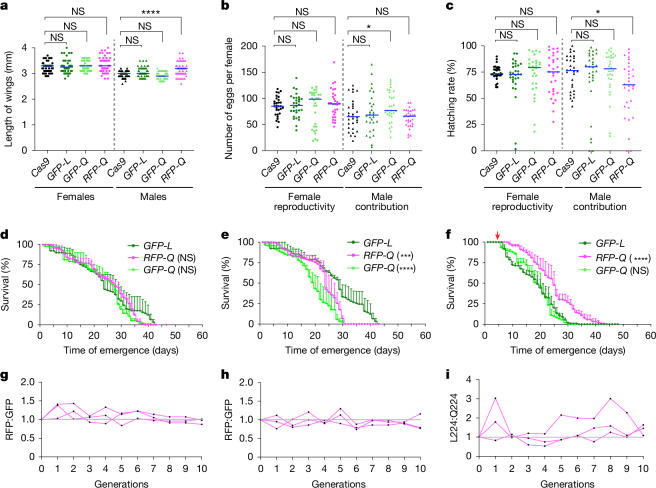
Characterization of the fitness of *FREP1* allelic variants. **a**, Length of wings. Cas9, *vasa*-Cas9; *GFP-L*, *FREP1*^*GFP-L*^; *GFP-Q*, *FREP1*^*GFP-Q*^; *RFP-Q*, *FREP1*^*RFP-Q*^. **b**, Number of eggs. Each dot indicates egg numbers from a single female mosquito. **c**, Hatching rate of eggs counted from panel **b**. The columns with circles indicate females, and the triangles denote males; the blue bars indicate median value; *n* = 30 individual mosquitoes (**a**–**c**). Statistical significance was calculated with a one-way analysis of variance (ANOVA) multiple comparisons test, and adjusted *P* values are shown in Source Data. **d**–**f**, Lifespan of virgin females (**d**) and males maintained with a 10% sucrose diet (**e**), and of females maintained with a 10% sucrose diet after mating and blood feeding (**f**). The red arrow indicates blood meal applied (**f**). *n* = 3 biologically independent replicates were used for the lifespan test; statistical significance was calculated by Kaplan–Meier survivability analysis with pooled data from three biological replicates (**d**–**f**). Data are mean ± s.d. **g**,**h**, The number ratio of RFP:GFP-marked transgenic mosquitoes observed when crossing *FREP*^*RFP-Q*^ mosquitoes with *FREP*^*GFP-L*^ (**g**) or with *FREP*^*GFP-Q*^ (**h**). **i**, Ratio of allele frequencies between *FREP1*^*GFP-L*^ and *FREP1*^*GFP-Q*^ transgenic mosquitoes. *n* = 3 biological independent experiments shown by each line (**g**–**i**). Not significant (NS) *P* > 0.05, *0.01 < *P* < 0.05, ***0.0001 < *P* < 0.001 and ****P < 0.0001 (**a**–**f**). Source data

We assessed possible fitness costs associated with the *FREP1*^*Q*^ allele by quantifying the body size, fecundity and longevity of *FREP1*^*Q*^ versus *FREP1*^*L*^ mosquito strains (Fig. 2). We used wing length as a proxy for body size and *vasa*-Cas9 as an additional control, as the *FREP1*-transgenic lines were generated from the *vasa*-Cas9 strain. Thus, all three strains shared the same genetic background. We found that body size was comparable between the controls and the two *FREP1*^*Q*^ strains, except for a slight, but statistically significant, increase in *FREP1*^*RFP-Q*^ males. This difference may be attributed to stochastic sampling of a limited sample size and/or minor genetic variations (Fig. 2a). In terms of fecundity, all *FREP1* transgenic females displayed similar levels of fecundity to *vasa*-Cas9 females when crossed with WT males (Fig. 2b), whereas a modest increase in male contribution to reproductive success was observed for *FREP1*^*GFP-Q*^ males when crossed with WT females (Fig. 2b). Egg hatching rates were assessed 4 days after egg laying and, once again, no significant difference was noted among the compared strains except for a minor decrease when *FREP1*^*RFP-Q*^ males were crossed to WT females (Fig. 2c).

**Extended Data Fig. 3:**
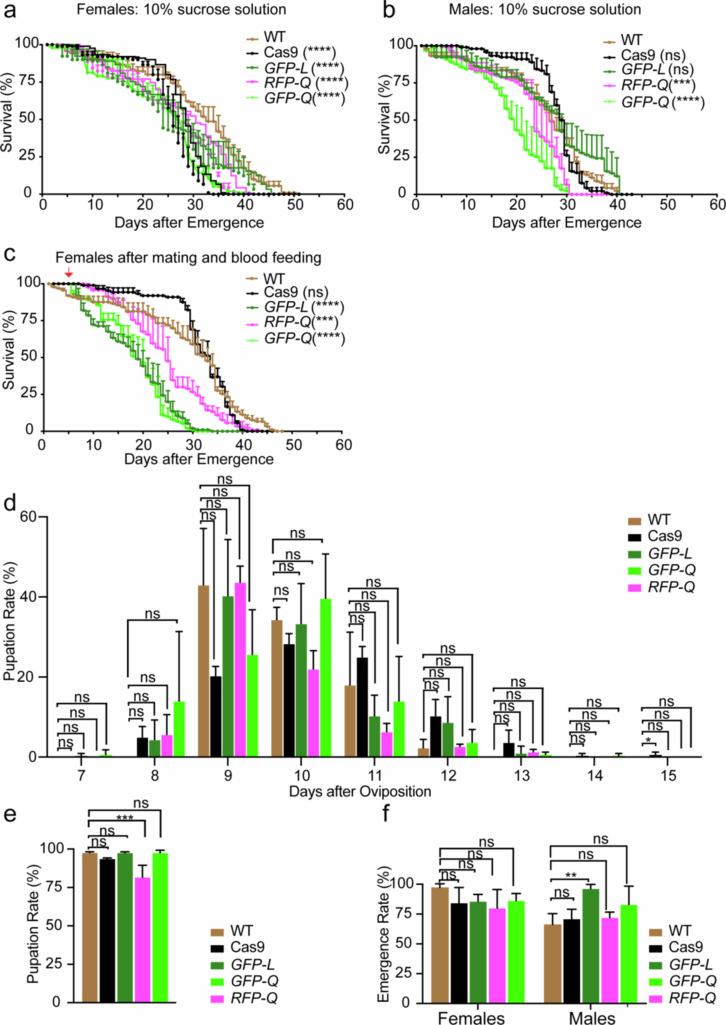
Life spans of three *FREP1* allelic variants compared to WT *An. stephensi* and *vasa*-Cas9 strains. **a**, Life span of females that were maintained with a 10% sucrose solution. **b**, Life span of males maintained on a 10% sucrose solution. **c**, Life span of females after mating and blood feeding. Red arrow indicates blood meal applied. The mean values calculated from three replicated cages are shown. In a-c, n = 3 biologically independent replicates, statistical significance was calculated by Kaplan-Meier survivability analysis with pooled data from three biological replicates. Error bars are mean ± S.D. The X-axis represents days after adult emergence, and the Y-axis indicates the percentage of surviving adults. **d**, Pupation time of *FREP1* transgenic lines. **e**, Pupation rate. **f**, Adults emergence rate of female and male pupae. For d-f, statistical significance was calculated with an Ordinary One-way ANOVA multiple comparison tests and adjusted *p*-values shown in the Source Data. n = 3 biologically independent replicates for the experiments, and error bars indicate mean ± S.D., with ns: no significance, *P* > 0.05, *: 0.01 < *P* < 0.05, **: 0.001 < *P* < 0.01. Cas9: *vasa*-Cas9, *GFP-L*: *FREP1*^*GFP-L*^; *GFP-Q*: *FREP1*^*GFP-Q*^; *RFP-Q*: *FREP1*^*RFP-Q*^. Source data

We also examined the potential fitness costs of *FREP1*-transgenic mosquitoes with respect to lifespan using the *FREP1*^*GFP-L*^ congenic line as the control to evaluate whether altering the L224 codon (*FREP1*^*GFP-L*^ versus *FREP1*^*GFP-Q*^) imposed any fitness cost. In addition, we tested whether any fitness differences resulted from the insertion of alternative fluorescence markers at the same intronic site (*FREP1*^*RFP-Q*^ versus *FREP1*^*GFP-Q*^). This analysis revealed that the virgin females of both Q224 alleles exhibited similar lifespans compared with the *FREP1*^*GFP-L*^ control (Fig. 2d and Extended Data Fig. 3a). In the case of males, the *FREP1*^*GFP-L*^ strain had a more gradual mortality trajectory than the two *FREP1*^*Q*^ allelic variants, with the curve levelling off during later timepoints (Fig. 2e and Extended Data Fig. 3b). These modest differences in mortality may have arisen from yet uncharacterized biological functions of *FREP1* involved in male developmental processes or from unknown variations in experimental factors affecting the assay (Fig. 2e and Extended Data Fig. 3b). We also noted a minor, but statistically significant, difference in mortality between the two differentially marked Q224 transgenic lines, potentially due to distinct characteristics of the fluorescence markers (Fig. 2e). However, as described in more detail below, these two alleles maintained approximately constant frequencies when competing against each other in multi-generational cage experiments (Fig. 2h), suggesting the overall impact of such differences is most likely quite small. Also, *FREP1*^*GFP-Q*^ and *FREP1*^*GFP-L*^ females that were blood fed after mating displayed a slightly reduced longevity (compared with virgin females; Fig. 2d,f and Extended Data Fig. 3c). This effect was less pronounced in *FREP1*^*RFP-Q*^ mosquitoes, whereas the *FREP1*^*GFP-Q*^ and *FREP1*^*GFP-L*^ allelic variants exhibited comparable lifespan profiles (Fig. 2f and Extended Data Fig. 3). These parallel comparisons revealed only a significant reduction in male lifespan of the *FREP1*^*GFP-Q*^ allele compared with the *FREP1*^*RFP-Q*^ and *FREP1*^*GFP-L*^ alleles (Fig. 2d–f). In addition, all three transgenic strains exhibited a similar significant longevity decrease relative to WT and *vasa*-Cas9 controls (Extended Data Fig. 3a–c). This modest difference may reflect a fitness cost associated with the *FREP1* transgenic insertion; however, such effects do not appear to impact the multi-generational experiments, in which the transgenic Q224-linked cassette competes effectively with a WT L224 allele (Extended Data Fig. 9, see below).

In addition, we examined pupation and adult emergence rates, again with only minor variations associated in *FREP1* transgenic mosquitoes (Extended Data Fig. 3d–f). Overall, we did not note any consistent significant fitness costs across all parameters, suggesting that the observed sporadic fitness costs may arise from stochastic sampling of limited populations. We conclude that changing the codon for a single amino acid (CTA to CAA) results in only modest fitness differences between the *FREP1*^*L*^ and *FREP1*^*Q*^ congenic allelic variants, which may reflect small differences between the transgene insertions and/or stochastic variations between experiments.

We further assessed the relative fitness of the three *FREP1* strains by conducting stringent multi-generational competition experiments between *FREP1* alleles in freely mating populations. Cages were initially seeded with transheterozygotes, resulting in a 50% initial frequency for each allele (Fig. 2g–i). Allele frequencies were then scored over 10 consecutive generations (for example, *FREP1*^*RFP-Q*^ versus *FREP1*^*GFP-L*^ or *FREP1*^*RFP-Q*^ versus *FREP1*^*GFP-Q*^) by tallying the ratio of the mosquitoes carrying the distinguishing fluorescence markers at each generation (Fig. 2g,h). We observed an initial pattern of seemingly random fluctuations in allelic ratios during the first few generations, followed by a stable trajectory that gradually approached unity (Fig. 2g,h), indicating that the competing lines had comparable fitness. In addition, we evaluated competition between the *FREP1*^*GFP-L*^ and *FREP1*^*GFP-Q*^ strains, which carry the same fluorescent marker, using targeted deep sequencing to determine the relative frequency of each allele in each generation (Fig. 2i). Aside from occasional late outlier fluctuations for the paired *GFP* lines (Fig. 2i), we observed no significant difference in relative competitiveness between these two *FREP1* alleles, confirming the findings described above that there are modest, if any, consequential differences between the various tested transgenic strains.

## *FREP1*^*Q224*^ mosquitoes are parasite resistant

**Fig. 3:**
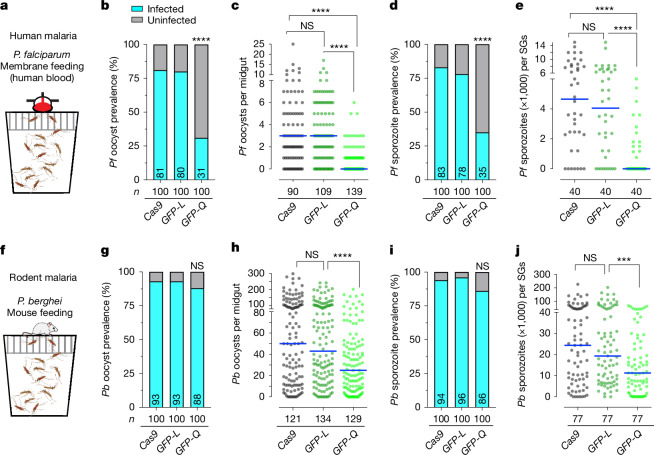
*FREP1*^*Q*^ mosquitoes are resistant to both *P. falciparum* and *P. berghei* infection. **a**, A standard feeding membrane assay for *P. falciparum* infection in the mosquito population including females and males with gametocyte (NF54)-infected human blood. **b**,**c**, Infection prevalence (**b**) and infection intensities (**c**) of *P. falciparum* (NF54) oocyst loads in the midguts of *vasa*-Cas9 control and two *FREP1* transgenic mosquitoes (*FREP1*^*GFP-L*^ and *FREP1*^*GFP-Q*^) at low gametocytaemia (0.08%) at 8 days post-infection (dpi). **d**,**e**, Infection prevalence (**d**) and infection intensities (**e**) of *P. falciparum* (NF54) sporozoites in salivary glands (SGs) at 15 dpi. **f**, Mosquito population with females and males was infected with *P. berghei* through mouse (no bias on gender) feeding. The schematics in panels **a**,**f** were created using BioRender (https://biorender.com). **g**–**j**, Prevalence and infection intensities tabulated for *P. berghei* oocysts at 12 dpi (**g** and **h**) and sporozoites at 21 dpi (**i** and **j**) at high infection level. Each single dot represents the number of parasites in an individual dissected midgut or one pair of salivary glands. The horizontal lines denote median values (**c**,**e**,**h**,**j**). *n* = 3 biological replicates, and the final pooled numbers are indicated in Tables 1 and 2. *n* denotes the number of tested individual mosquitoes. A two-tailed Mann–Whitney *U*-test was used to determine statistical significance for infection intensities, and a Fisher’s exact test was used for infection prevalence. NS *P* > 0.05, ***0.0001 < *P* < 0.001 and ****P < 0.0001. Source data

**Extended Data Fig. 4:**
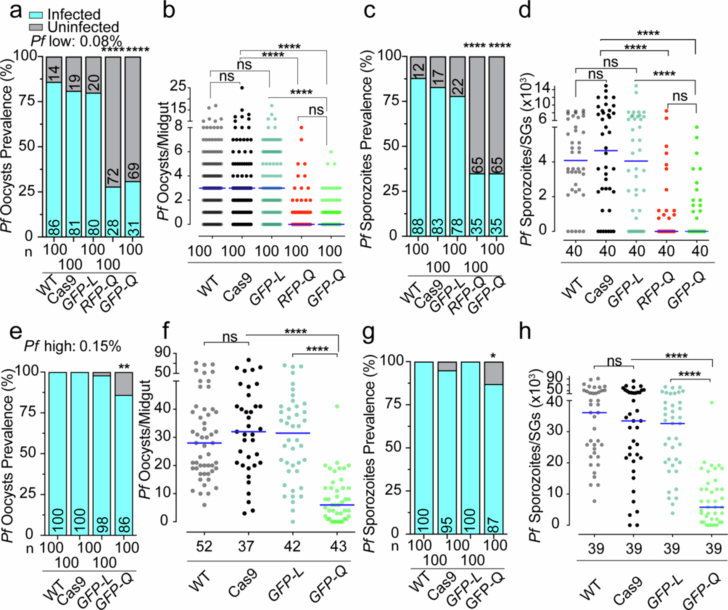
*FREP1*^*Q*^ mosquitoes are resistant to *P. falciparum* at both low and high parasite infection levels. **a**, Prevalence of *P. falciparum* (NF54) oocysts in response to infections at low infection level (0.08% gametocytemia). **b**, Infection intensities of *P. falciparum* (NF54) oocysts in WT, *vasa*-Cas9, and three *FREP1* transgenic mosquitoes at a low infection intensity. **c**, **d**, Prevalence (**c**) and infection intensities (**d**) of *P. falciparum* (NF54) sporozoites at a low infection intensity. **e-h**, Prevalence and infection intensities with *P. falciparum* (NF54) oocysts (**e** and **f**) and sporozoites (**g** and **h**) at a high infection intensity (0.15% gametocytemia). At least 40 successfully infected female mosquitoes were randomly selected and dissected for counting oocysts in the midgut or sporozoites in the salivary glands. All mosquitoes counted from the same line were pooled for display, with each single dot representing the parasite load from the individual dissected midgut or a pair of salivary glands. The horizontal lines in panels **b**, **d**, **f**, and **h** denote median values. Two-tailed Mann-Whitney U test was used to calculate *P* values. n indicated the total pooled number of individual mosquito midguts or salivary glands measured. All parasite challenge assays were conducted with at least three independent biological replicates. Cas9: *vasa*-Cas9, *GFP-L*: *FREP1*^*GFP-L*^; *GFP-Q*: *FREP1*^*GFP-Q*^; *RFP-Q*: *FREP1*^*RFP-Q*^. The statistical details were included in Supplementary Tables 1–4. Source data

Next, we assessed whether the *FREP1*^*Q*^ allele reduced infection of *A. stephensi* by the major human malaria parasite *P. falciparum* through membrane feeding on parasite gametocytes mixed with human red blood cells and serum^6,13^ (Fig. 3a). We then measured both oocyst infection prevalence (percentage of mosquito midguts with developed parasite oocysts) and intensity (the number of oocysts per midgut) 8 days after feeding on both low and high gametocytaemia blood^6^. At low gametocyte concentration (0.08% gametocytaemia), infection intensities typical for mosquitoes in the field, we observed a significant reduction in infection prevalence in the *FREP1*^*GFP-Q*^ strain, decreasing from around 80% in control (*vasa-*Cas9 and *FREP1*^*GFP-L*^) mosquitoes to approximately 30% (Table 1, Fig. 3b, Extended Data Fig. 4a–d and Supplementary Table 1). Infection intensity, measured as the median number of oocysts per midgut, was also strikingly decreased from 3 to 0 in the control and the *FREP1*^*GFP-Q*^ strains, respectively (Table 1, Fig. 3c and Extended Data Fig. 4b). Similarly, at a high gametocyte concentration (0.15% gametocytaemia), we observed significant decreases in infection prevalence (dropping from 98% in *FREP1*^*GFP-L*^ control to 86% in the *FREP1*^*GFP-Q*^ line) and intensity (decreasing from a median of about 32 oocysts per midgut in *FREP1*^*GFP-L*^ control to less than 10 oocysts per midgut for the *FREP1*^*GFP-Q*^ line; Extended Data Fig. 4e,f and Supplementary Tables 1 and 3). By contrast, oocyst infection intensities and prevalence were comparable between *FREP1*^*GFP-L*^ and *vasa*-Cas9 controls, indicating that only mosquitoes carrying the *FREP1*^*Q*^ allele were robustly resistant to *P. falciparum* infection (Table 1 and Fig. 3b,c).

**Table 1:** *P. falciparum* infection at a low gametocytaemia level (0.08%)

	Oocysts			Sporozoites		
	*vasa*-Cas9	*FREP1*^*GFP-L*^	*FREP1*^*GFP-Q*^	*vasa*-Cas9	*FREP1*^*GFP-L*^	*FREP1*^*GFP-Q*^
*n*	90	109	139	40	40	40
Mean	3.8	3.5	0.6	5,339	4,336	950
Median	3	3	0	4,650	4,050	0
Mann–Whitney *U*-test (two-tailed) *P* value	Control	NS	<0.0001	Control	NS	<0.0001
Prevalence (%)	81.1	79.8	31.7	82.5	77.5	35.0
Fisher’s exact *P* value	Control	NS	<0.0001	Control	NS	<0.0001
Range	0–25	0–17	0–6	0–15,000	0–15,000	0–6,000
Reduction in median (%)	–	Control	100	–	Control	100

NS, *P* > 0.05.

We also quantified the number of sporozoites present in the salivary glands to assess the efficacy of malaria transmission blocking, as the sporozoite load in salivary glands is directly linked to the ability of a mosquito to transmit malaria parasites^36,37^. We observed an approximately fivefold reduction in the median number of salivary gland sporozoites from more than 4,650 sporozoites per salivary gland pair for *vasa*-Cas9 and 4,050 sporozoites for *FREP1*^*GFP-L*^ controls compared with a median of zero sporozoites in *FREP1*^*GFP-Q*^ salivary glands (Fig. 3d,e and Extended Data Fig. 4d). Similarly, at high infection intensities, large reductions in sporozoite burdens were observed in *FREP1*^*Q*^ versus *FREP1*^*L*^ strains (Extended Data Fig. 4g,h and Supplementary Table 2). Together, these findings support the hypothesis that mosquitoes carrying the *FREP1*^*Q*^ allele are highly refractory to infection by *P. falciparum* parasites, as indicated by measures of parasite prevalence, median number of oocysts and total sporozoite loads. The parallel observation of the degree of reduction in oocyst numbers and depletion of later-stage sporozoites in *FREP1*^*Q224*^ mosquitoes compared with the congenic *FREP1*^*L224*^ strain suggests that the protective *FREP1*^*Q224*^ allele inhibits parasite development at early pre-oocyst stages.

The FREP1 protein has been identified as a broad-spectrum target for transmission-blocking vaccines that target malaria parasites in the mosquito vector^2,38^. However, the transmission target epitopes in these diverse studies remain uncharacterized. Thus, we fed *FREP1*^*GFP-Q*^ and control lines (*vasa*-Cas9 and *FREP1*^*GFP-L*^) on mice infected with the divergent rodent parasite *P. berghei* (WT, ANKA 2.34; Fig. 3f). We found again that only the *FREP1*^*GFP-Q*^ transgenic mosquitoes showed a significant reduction in high-intensity *P. berghei* infection at both the oocyst and the sporozoite stages (for example, the median infection intensity dropped from 43 to 25 oocysts per midgut, respectively, in the *FREP1*^*GFP-L*^ versus *FREP1*^*GFP-Q*^ strains; Table 2 and Fig. 3g–j). In these experiments, we did not observe a significant reduction in oocyst prevalence in infected *FREP1*^*GFP-Q*^ mosquitoes, but this was presumably due to *P. berghei* not being a natural parasite of *A. stephensi* and the typically high infection intensities that *P. berghei* achieves in this unnatural vector.

**Table 2:** *P. berghei* infection at a high gametocytaemia level (0.15%)

	Oocysts			Sporozoites		
	*vasa*-Cas9	*FREP1*^*GFP-L*^	*FREP1*^*GFP-Q*^	*vasa*-Cas9	*FREP1*^*GFP-L*^	*FREP1*^*GFP-Q*^
*n*	121	134	129	77	77	77
Mean	73.9	63.3	36.1	37,263	36,134	15,995
Median	50	43	25	24,360	19,320	11,250
Mann–Whitney *U*-test (two-tailed) *P* value	NS	Control	<0.0001	NS	Control	0.0007
Prevalence (%)	94.2	94.0	89.1	93.5	97.4	85.7
Fisher’s exact *P* value	NS	Control	0.3106	NS	Control	0.0093
Range	0–299	0–242	0–202	0–226,800	0–203,280	0–68,040
Reduction in median (%)	–	Control	41.9	–	Control	41.8

NS, *P* > 0.05.

We also performed parasite challenge assays with *FREP1*^*L224*^/*FREP1*^*Q224*^ transheterozygotes to evaluate whether the *FREP1*^*Q224*^ allele in a heterozygous condition could also confer resistance to the parasite infection. In this case, we did not detect any significant resistance to either *P. falciparum* or *P. berghei* infection, consistent with the results reported previously in *A. gambiae*^3^. We conclude that the *FREP1*^*Q*^ allele in *A. stephensi* confers a broad-spectrum parasite-refractory phenotype when homozygous.

## A linked *FREP1*^*Q*^ allelic-drive cassette

**Fig. 4:**
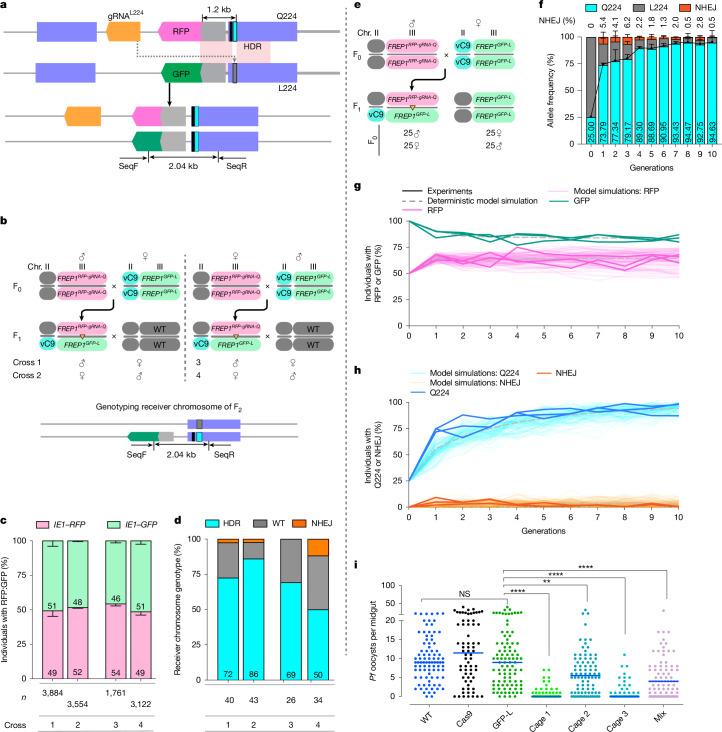
Disseminating the *FREP1*^*Q224*^-refractory allele by the linked allelic-drive system. **a**, Linked allelic-drive scheme (top). The grey lines denote genomic DNA; the purple boxes indicate exons; the narrow cyan rectangle denotes the Q224 residue; the narrow grey rectangle indicates the WT L224 residue; the narrow black rectangle denotes the non-synonymous cleavage-resistant residue; and the light pink boxes indicate chromosomal homology between donor and receiver chromosomes. Allelic replacement of the L224 codon on the receiver chromosome with the Q224 edit is also shown (bottom). SeqF and SeqR refer to primers for receiver chromosome-specific amplification. **b**, Cross schemes and genotyping primer set for controlled three-generation pair-mating crosses. Chr. chromosome. **c**, Genotyping of the F_2_ progeny using fluorescence markers. *n* = 3 biologically independent crosses. Data indicate mean ± s.d. *n* Denotes the number of total F_2_ progeny counted. **d**, L224 to Q224 conversion rate quantified by Sanger sequencing. *n* Denotes the number of F_2_ progeny tested. **e**, Cross scheme for generating transheterozygous used for cage trial seeding. **f**, Inferred allelic frequencies across 10 generations. Q224 is in cyan, L224 in grey and NHEJ in orange. Three cages were run as biological replicates. Data indicate mean ± s.d. The black line shows the profile of mean Q224 allele frequencies. **g**, Frequency of individuals carrying each fluorescent marker. **h**, Allelic frequency in the cage population. The light coloured lines show the experimental data, and the faint lines are 100 stochastic model simulations (**g**,**h**). The dashed grey lines denote deterministic model simulations. **i**, *P. falciparum* (NF54) oocyst loads in the midguts of controls (WT, *vasa*-Cas9 and *FREP1*^*GFP-L*^) and generation of 11 cage trial mosquito populations (three cages tested separately and mixed) fed on low gametocytaemic (0.08%) blood. The horizontal lines denote median values (**i**). A two-tailed Mann–Whitney *U*-test was used to determine statistical significance for infection intensities, and a Fisher’s exact test for infection prevalence. NS *P* > 0.05, **0.001 < *P* < 0.01 and ****P < 0.0001. Source data

We wondered whether it might be possible to use a gene-drive system to promote super-Mendelian transmission of the parasite-resistant *FREP1*^*Q224*^ allele relative to the infection-susceptible *FREP1*^*L224*^ allele. We therefore designed a linked allelic-drive cassette (*FREP1*^*RFP-gRNA-Q*^; Fig. 4a) carrying the RFP fluorescent marker, the Q224 edit and gRNA^L224^, the last selectively targeting the parasite-permissive *FREP1*^*L*^ allele. This gRNA^L224^-bearing gene-editing cassette was inserted at the same genomic site in the second intron as the RFP-marked and GFP-marked transgenic elements described above (Figs. 1a and 4a). We hypothesized that when combined with Cas9, gRNA^L224^ would convert the L224 residue on the homologous chromosome to the Q224 residue via homology-directed repair in developing germ cells (Fig. 4a).

**Extended Data Fig. 6:**
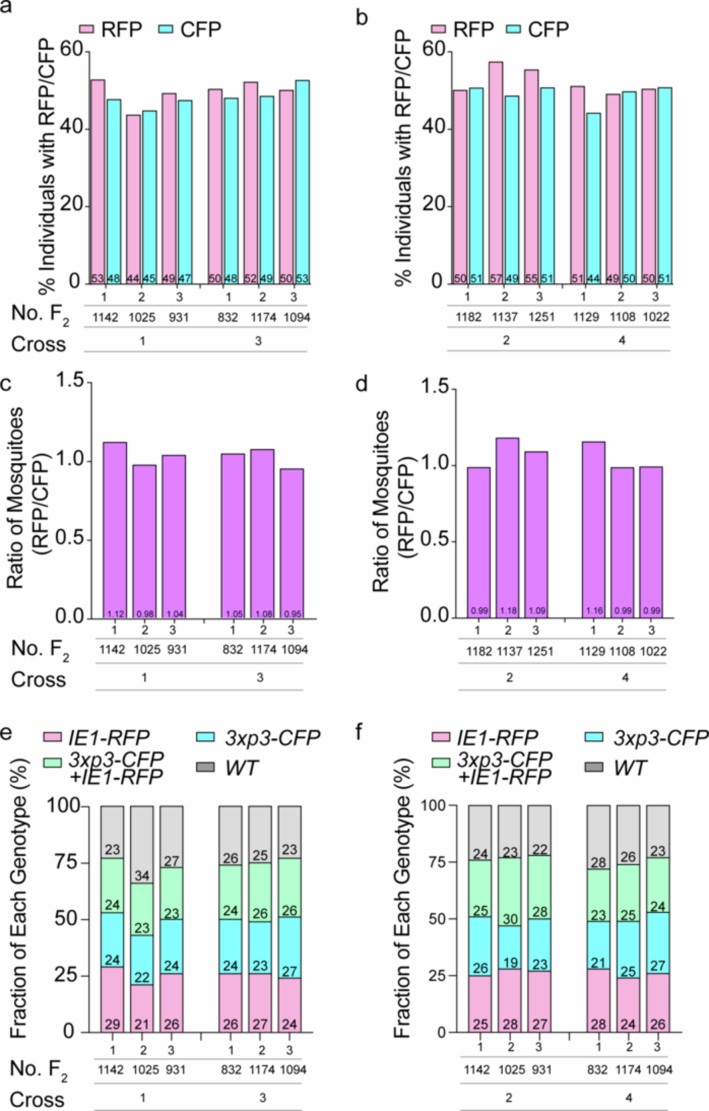
Phenotype of F_2_ progeny generated from crosses between *FREP1*^*RFP-Q*^ and *vasa*-Cas9 strains. **a, b**, F_2_ progeny phenotyping results display the fraction of total F_2_ progeny carrying RFP or CFP fluorescence: **a**, crosses using F_1_ trans-heterozygous males. **b**, Crosses using F_1_ trans-heterozygous females. Data display the results from two reciprocal F_0_ crosses: F_0_ *vasa*-Cas9 males are crossed with *FREP1*^*RFP-Q*^ females or reciprocally, F_0_ *vasa*-Cas9 females are crossed with *FREP1*^*RFP-Q*^ males. Fractions of each phenotype (%) are indicated beneath each bar. Pink bars = RFP fluorescence and cyan bars = CFP. Three replicates were conducted for each cross. **c, d**, The ratio of population size between RFP and CFP phenotypes from F_2_ progeny. **e, f**, Fractions of indicated phenotypes in F_2_ progeny. Phenotyping was conducted by counting the presence of fluorescence markers either with whole-body RFP (marks *FREP1*^*RFP-gRNA-Q*^ chromosome with *IE1-RFP*) or eye-specific CFP (marks *vasa*-Cas9 with *3xP3-CFP*). The total number of counted F_2_ progeny and the detailed cross information are listed at the bottom table. Source data

**Extended Data Fig. 7:**
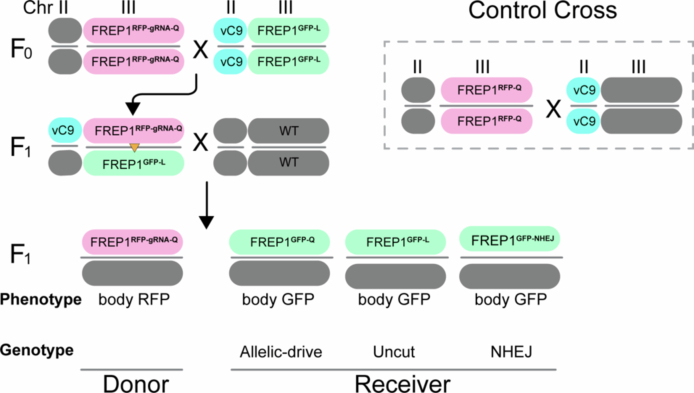
Cross scheme. vC9 stands for *vasa*-Cas9, NHEJ: Non-homologous end joining.

**Extended Data Fig. 8:**
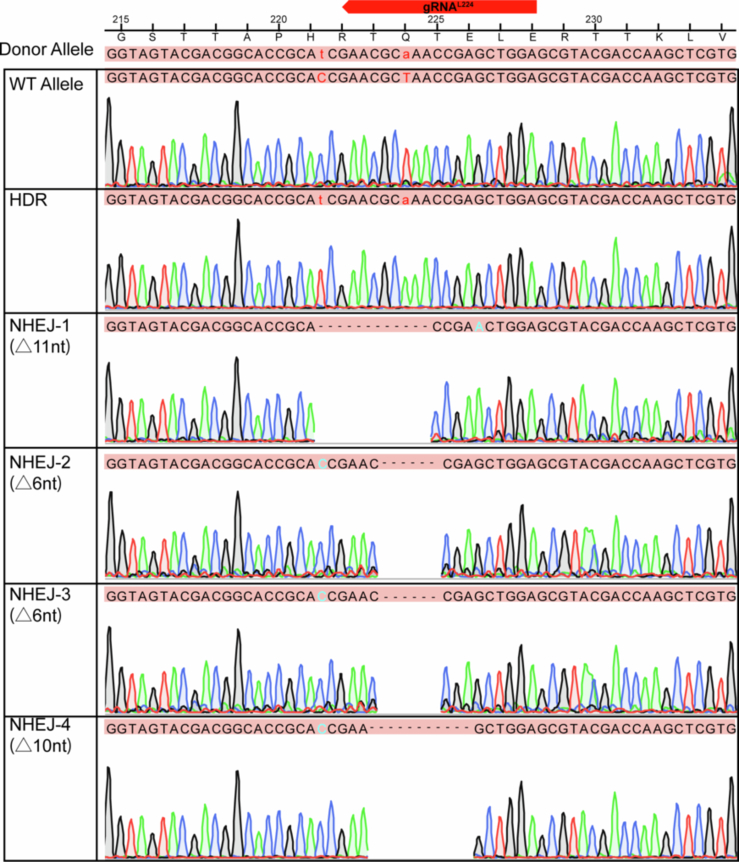
DNA sequencing traces illustrating germline repair events that occurred on the target chromosome in the germline. Traces display DNA sequences flanking the gRNA^L224^ cleavage point. Single F_2_ progeny carrying only *vasa*-Cas9 (*3xP3-CFP*) were used for targeted amplification and Sanger sequencing. Three categories of DSB repair events are displayed: WT, HDR, and NHEJ (11nt, 6nt, and 10nt deletions, respectively). The top line shows the gRNA^L224^ target marked with red arrowheads, followed by translated amino acids. DNA sequences flanking the gRNA^L224^ target show the repair template or sequences from the donor chromosome at *FREP1 loci*. The T->A and C->T edits were shown with red letters.

We first tested the above hypothesis in a three-generation controlled pair-mating scheme (Fig. 4b) using an unlinked source of *vasa*-Cas9 (Extended Data Figs. 6 and 7). In these experiments, we used the above constructed *FREP1*^*GFP-L*^ chromosome as the target allele, using distinguishable fluorescence markers (*IE1-RFP*: *FREP1*^*RFP-gRNA-Q*^ and *IE1-GFP*: *FREP1*^*GFP-L*^) to track the chromosomes (Fig. 4a). Transheterozygous *FREP1*^*RFP-gRNA-Q*^/*FREP1*^*GFP-L*^ F_1_ mosquitoes (either males or females) were crossed to a WT *A. stephensi* strain, and the phenotypes and genotypes of the resulting F_2_ progeny were tabulated (Fig. 4b). We scored the fraction of the two distinguishing fluorescence markers in F_2_ progeny and observed approximately equal inheritance of donor versus target chromosomes, confirming standard Mendelian inheritance of the *FREP1*^*RFP-gRNA-Q*^ gene cassette (gRNA^Intron^ used for insertion of the cassettes was not included in this cassette; Fig. 4c). We also assessed the frequency of allelic conversion (L224 > Q224) on the differentially marked *FREP1*^*GFP-L*^ target chromosome (that is, GFP^+^RFP^–^ F_2_ progeny) by performing allele-specific amplification (PCR) using a 5′ primer (SeqF) complementary to the GFP, followed by Sanger sequencing of target chromosome-derived amplicons (Fig. 4b,d). This analysis revealed robust gene conversion frequencies of the target chromosome inherited both paternally (72% conversion, or 86% overall Q224 allele frequency) and maternally (86% conversion, 93% overall Q224 allele frequency) when Cas9 was provided from F_0_ grandmothers (Fig. 4d). Somewhat lower conversion frequencies (69% paternal and 50% maternal, or 84.5% and 75% of total alleles, respectively) were observed when Cas9 was inherited from grandfathers (Fig. 4d). In addition, a modest frequency of non-homologous end-joining (NHEJ) alleles was observed (ranging from 0% to 11.8% of receiver alleles depending on the crossing scheme). Half of such NHEJ mutations derived from the grandfather crosses were out-of-frame as assessed by DNA Sanger sequencing (Extended Data Fig. 8), whereas mutant alleles arising from grandmother crosses all had large, presumably non-functional insertions. The remaining predominant class of target alleles was WT (L224; Fig. 4d), which we inferred were either precisely repaired or uncut. Thus, under these conditions, we observed a high frequency of target conversion from the *FREP1*^*L*^ to the *FREP1*^*Q*^ allele (approximately 70% on average, ranging from 50% up to 86%) with only a modest fraction of target site mutations being generated, the latter consisting predominantly of loss-of-function *FREP1* alleles that presumably would incur substantial fitness costs.

## The *FREP1*^*Q224*^ allele drives in populations

We further assessed the linked allelic-drive element by testing whether it could sustain efficient drive of the *FREP1*^*Q224*^ allele in multi-generational cages of freely mating mosquitoes. We conducted triplicate non-overlapping small laboratory population cage experiments initiated by seeding transheterozygous mosquitoes carrying the *vasa*-Cas9; *FREP1*^*RFP-gRNA-Q224*^ gene cassette and the *FREP1*^*GFP-L*^ target allele into homozygous *FREP1*^*GFP-L*^ populations at a 1:3 allelic ratio (Fig. 4e–h). The fraction of mosquitoes carrying the linked allelic-drive (pink lines) rose from 50% in the first generation to approximately 64% and remained constant through the 10th generation (Fig. 4g; see modelling section below). Reciprocally, mosquitoes carrying the receiver *FREP1*^*GFP-L*^ allele (green lines) dropped to a steady-state level of approximately 83% (Fig. 4g). These results are consistent with the data presented in Fig. 2g–i, where competing congenic *FREP1*^*RFP-Q*^ and *FREP1*^*GFP-L*^ alleles in multi-generational cages displayed approximately equal fitness. In addition, the observation that the *FREP1*^*RFP-gRNA-Q*^ and *FREP1*^*GFP-L*^ alleles co-existed over several generations (see below) further supports our hypothesis that *FREP1*^*Q224*^ and *FREP1*^*L224*^ alleles have similar fitness.

In each generation, we also genotyped 50 randomly selected mosquitoes by next-generation sequencing (NGS) for their *FREP1*^*L224/Q224*^ genotypes. We selectively amplified the gRNA target site from the *FREP1*^*GFP-L*^ receiver chromosome using GFP-specific primers and performed NGS sequencing. We inferred the allelic frequency in the total population on the basis of the observed conversion rates weighted by the fraction of marked donor versus receiver chromosomes scored by their fluorescence phenotype (Fig. 4f,h). The frequency of the *FREP1*^*Q224*^ allele increased rapidly from its initial 25% seeding percentage to more than 90% introduction over the course of 10 generations (Fig. 4f,h).

In all cages, only a modest fraction of NHEJ alleles were generated, which dropped from an initial average of 5.4% at generation 1 to less than 0.5% by generation 10, showing a trend of gradual elimination (Fig. 4f,h). This progressive loss of NHEJ alleles is consistent with potential fitness costs being associated with loss-of-function *FREP1* mutations. These results support the hypothesis that the linked allelic-drive system as configured sustains efficient allelic drive of the protective *FREP1*^*Q224*^ allele without producing a significant fraction of interfering alternative NHEJ alleles.

**Extended Data Fig. 9:**
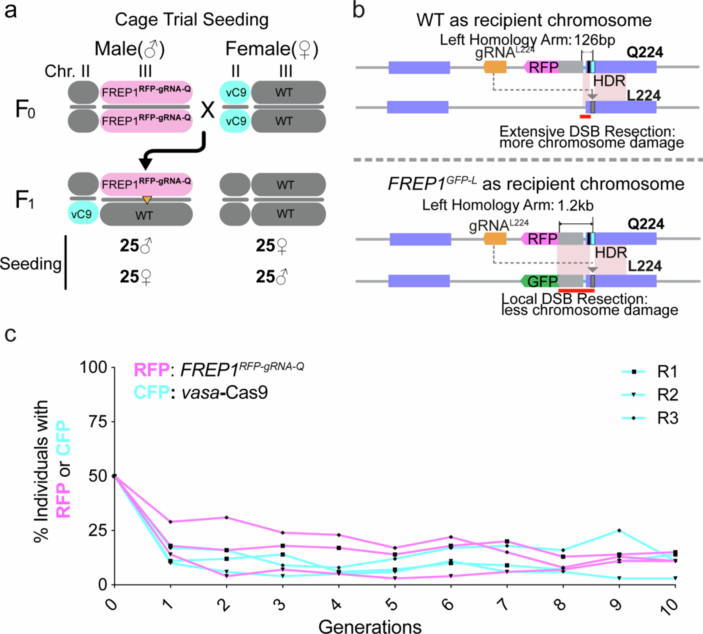
Non-overlapping multi-generational cage trial with a WT population. **a**, Genetic crossing scheme used to generate F_1_ trans-heterozygous for seeding the cage experiments. **b**, Comparison of homology pairing between donor and receiver chromosomes in different linked allelic-drive configurations. The donor driving cassette is placed either *in trans* to the WT L224 allele (top panel) or to the *FREP1*^*GFP-L*^ allele as shown in Fig. 4e–h (bottom panel). Pink boxes depict regions of the homology pairing on the intronic side of the gRNA cut site that are shared between the donor and receiver chromosomes. On the intronic side of the WT L224 allele, there is only a shortened homology of 126 bp (indicated by the narrow red bar) shared with the drive cassette resulting in frequent error-prone DSB repair and generation of damaged target alleles. In contrast, the congenic experiments presented in Fig. 4 involve a much longer (>1.2 kb, indicated by wide red bar) span of homology between the two parental chromosomes. Gray lines: genomic DNA at *FREP1* locus, purple boxes: exons, cyan narrow rectangle: Q224 residue, gray narrow rectangle: WT L224 residue, black narrow rectangle: non-synonymous cleavage-resistant residue. **c**, The frequency of individuals carrying each fluorescence marker phenotype (RFP for the gRNA cassette and GFP for the Cas9 cassette) is plotted over 10 generations. The frequency of both the *FREP1*^*RFP-gRNA-Q*^ (pink lines) and *vasa*-Cas9 (cyan lines) alleles drops rapidly in the first generation due to a high rate of target chromosome damage and consequent lethality of a significant fraction of such progeny (see above), but then stabilizes as the frequency of progeny carrying both the gRNA and Cas9 encoding cassette drops to a low level (1–2%, see Source Data). Three replicate cages (R1-R3) were run at the same time under the identical conditions. Source data

We also tested the performance of the linked *FREP1*^*RFP-gRNA-Q*^ drive in the context of the WT *FREP1*^*L224*^ allele, for which there was only limited homology on the intronic side of the gRNA cut side (126 bp between the donor and target chromosomes, compared with more than 1.2 kb of homology in the congenic configuration shown in Fig. 4e–h; Extended Data Fig. 9a,b). In this case of one-sided homology mismatch, a significant fraction of cleavage events resulted in damage to the target chromosome in both germline and somatic cells, leading to the lethality of a substantial significant fraction of individuals carrying both the gRNA^L224^ and the Cas9 transgenes, a phenomenon that has been well-documented in previous studies^39^. Such a drive configuration could, in principle, be used for localized allelic-drive applications (see Discussion). This experimental configuration also revealed that following an initial abrupt reduction in the frequency of the drive allele (as a consequence of the lethality mentioned above), the gRNA-bearing element remained at an approximately constant frequency for over 10 generations. Thus, the transgenic insertion element can compete effectively with respect to both WT and potentially generated functional NHEJ alleles, despite the observation in Extended Data Fig. 3c that post-blood-fed females exhibited a modest reduction in survival compared with WT.

Given the high level of final introduction of the *FREP1*^*Q*^ allele in all cages, we next tested whether these populations could suppress parasite infection. We conducted parasite challenge assays by feeding mosquitoes collected from cages at generation 11 on a *P. falciparum* gametocyte concentration that would produce a low infection intensity (Fig. 4i). Mosquitoes collected separately from all three population cages, or mixed together, demonstrated robust parasite suppression (the median infection intensity dropped from 9 to 0, 5.5 and 0 oocysts per midgut in cages 1, 2 and 3, respectively; Table 3 and Fig. 4i). These data strongly support the hypothesis that the *FREP1*^*Q*^ allelic variant can spread sufficiently to suppress parasite infection in final target mosquito populations.

**Table 3:** *P. falciparum* infection at a low gametocytaemia level (0.08%) with cage trial populations

	WT	*vasa*-Cas9	*FREP1*^*GFP-L*^	Cage 1	Cage 2	Cage 3	Cage mix
*n*	89	68	95	62	84	47	83
Mean	9.8	12.6	10.0	0.9	6.3	1.5	5.2
Median	9.0	11.5	9.0	0	5.5	0	4.0
Mann–Whitney *U*-test (two-tailed) *P* value	Control	–	NS	<0.0001	0.001	<0.0001	<0.0001
Prevalence (%)	97.8	89.7	95.8	33.9	83.3	42.6	75.9
Fisher’s exact *P* value	Control	0.033	0.6827	<0.0001	0.0004	<0.0001	<0.0001
Range	0–22	0–40	0–40	0–7	0–32	0–11	0–30
Reduction in median (%)	Control	–	0	100	38.9	100	55.6

NS *P* > 0.05.

## Mathematical modelling of drive dynamics

As a complement to the experiments described above, we also conducted Bayesian mathematical modelling to infer potentially hidden parameters that were not readily extracted from pair-mating or multi-generational experiments, such as subtle potential fitness costs or selective advantages of particular genotypes (Fig. 4g,h and Supplementary Tables 6–11).

A key inference from the model fitting was that the observed proportions of homozygous GFP mosquitoes (Source Data) in the first generation (F_1_) were consistently lower than expected (approximately 0.56 according to the model) assuming simple random assortment and an average rate of gene conversion of approximately 0.7 (Fig. 4d and Source Data). We hypothesized that the rapid rise of the linked allelic-drive might result from lethal sterile mosaicism, a process that we have previously described^8,10^. This process eliminates progeny homozygous for the *FREP1*^*GFP-L*^ allele that also inherit Cas9–gRNA complexes (transmitted maternally), and thus lack the repair template (*FREP1*^*RFP-gRNA-Q*^ allele). In these individuals, Cas9–gRNA^L224^ cleavage of both *FREP1*^*GFP-L*^ alleles could lead to the generation of homozygous loss-of-function alleles in many somatic cells phenocopying homozygous null *FREP1* mutants, which have severely reduced viability and fertility. As discussed further below, the overall drive kinetics can largely be accounted for by a combination of several factors including: (1) substantial allelic conversion (average of approximately 60%); (2) severe fitness costs levied on *FREP1*^*L224*^ homozygotes in the presence of Cas9–gRNA^L224^ complexes; (3) generation of only a modest number of NHEJ alleles (particularly of functional cleavage-resistant alleles); and (4) comparable fitness of the *FREP1*^*RFP-gRNA-Q*^ and *FREP1*^*GFP-L*^ alleles.

## Discussion

### *FREP1*^*Q*^ suppresses parasite infection

Parasite challenge assays conducted in this study using congenic *A. stephensi* strains carrying either the *FREP1*^*Q224*^ or *FREP1*^*L224*^ allele demonstrated that the *FREP1*^*Q224*^ allele selectively confers potent resistance to infection by two highly divergent malaria parasite species (*P. falciparum* and *P. berghei*), highlighting the broad protective effect of the *FREP1*^*Q224*^ allele. How the FREP1 protein facilitates the traversal of malaria parasites across the gut epithelium remains unknown; however, one hypothesis is that the L224 residue mediates a crucial interaction between FREP1 and yet-to-be-identified parasite surface factors^15,40^. This possibility, or alternatives involving differential FREP1 activities, merit examination in future studies.

Overall, our comparative studies demonstrated that: (1) the insertion of a selectable marker into the *FREP1* intron had minimal if any effect on the efficiency of parasite infection (that is, we observed similar intensities of parasite infection in the *FREP1*^*GFP-L*^ mosquitoes compared with the *vasa*-Cas9 L224 control strain); (2) all three *FREP1*-transgenic lines displayed only modest, if any, overall fitness differences based on parameters including body size, fecundity, longevity and success in undergoing metamorphosis and direct competition between congenic *FREP1* strains in multi-generational cages; and (3) the Q224 variant alone is sufficient in *A. stephensi* to confer resistance to infection by malaria parasites. To our knowledge, this is the first study in which genetic manipulation of a single amino acid of a mosquito factor has achieved robust inhibition of malaria parasite infection using gametocyte challenge levels equal to or greater than those that typically occur in the field.

*FREP1* has been investigated previously for its role in malaria parasite infection by using either RNAi-based silencing^2^ or CRISPR–Cas9-generated null mutants^13^. However, both of these systems have their limitations. The RNAi studies achieved partial and incomplete protein depletion, whereas null mutants, which displayed comparable parasite-refractory phenotypes to the single allelic replacements reported here, imposed high fitness costs^13^. Furthermore, the high degree of resistance to parasite infection conferred by the *FREP1*^*Q224*^ allele described in this study is comparable with that previously reported for a *FREP1*-null allele in *A. gambiae*^13^, indicating that this single amino acid change effectively eliminates FREP1 activity for parasite infection while leaving essential physiological functions of this protein in the mosquito intact. These findings in both *A. stephensi* and *A. gambiae* also point to FREP1 being a key protein required for parasite infection in two important *Anopheles* malaria vector species.

These high levels of parasite resistance conferred by the *FREP1*^*Q*^ and null alleles are akin to those provided by other infection blocking systems, such as gut-specific over-expression of the endogenous mosquito immune protective genes *Rel2* and *Akt*^17,41^ and parasite-blocking single-chain antibodies^6,21^. An important element of our current studies is the near fitness neutrality of the *FREP1*^*Q*^ allele in our extensive comparative tests of the congenic *FREP1*^*Q224*^ versus *FREP1*^*L224*^ strains. This fully functional phenotype of the *FREP1*^*Q224*^ allele contrasts with the severe reductions in fecundity and longevity previously reported for *A. gambiae FREP1*-null mutants^13^. In summary, our detailed results provide rigorous evidence supporting the hypothesis that the *FREP1*^*Q224*^ variant alone is sufficient to potently suppress parasite infection in *A. stephensi* and does so without appreciable cost to the host mosquito.

### Super-Mendelian propagation of *FREP1*^*Q*^

CRISPR-based gene-drive systems offer the potential for rapid and super-Mendelian dissemination of beneficial alleles through wild populations^42–44^. Efficient gene-drive systems have been developed in diverse organisms, including *Drosophila melanogaster*^45–48^, *Saccharomyces cerevisiae*^49^, *Anopheles* mosquitoes^8,10,50,51^, herpesviruses^52^ and *Escherichia coli*^53^. In addition, it is possible to combine canonical gene-drive elements with a second gRNA that selectively targets a non-preferred allele^42,43^. The linked allelic-drive cassette reported in this study represents an advance over previous allelic-drive systems^42,43,54^ by utilizing only a single gRNA that is closely linked to its cleavage site. This conjoined design avoids the free recombination between gene-drive cassette and functional drive-resistant insertions and deletions, which could otherwise be driven as ‘runaway’ alleles that potentially compete with the preferred allele for being driven into the population.

Mathematical modelling of the experimental data offered several insights into the factors that contribute to the overall efficiency of the drive process. In several aspects, this analysis parallels that of a conditionally self-eliminating drive system recently analysed in *Drosophila*^54^, in which a passively inherited drive cassette was inserted into the *voltage-gated sodium ion channel* (*vgsc*) locus. Extensive modelling, both accompanying the *vgsc* study and here for the *FREP1*^*Q224*^ drive, has revealed several synergistic factors leading to drive success: (1) substantial level of allelic-drive; (2) relatively low rates of NHEJ generation, and particularly of functional NHEJ alleles; (3) low fitness costs associated with the preferred driven allele; and (4) high fitness costs imposed on individuals inheriting only target alleles (for example, *FREP1*^*GFP-L*^ homozygotes in the current case) in combination with Cas9–gRNA (whether inherited genetically or transmitted maternally). The rationale behind the latter condition is that, in the presence of Cas9–gRNA complexes, individuals homozygous for *FREP1*^*L224*^ allele experience pervasive somatic mutagenesis of both copies of the L224 allele, leading to somatic mosaics in which many cells are homozygous for loss-of-function alleles of the *FREP1* gene. As there are very severe fitness costs (reduced viability and fertility) associated with *FREP1*-null alleles^13^, such lethal and/or sterile mosaic individuals are most likely to fail to survive or reproduce. On the basis of both the experimental data and in-depth supporting modelling, we conclude that the linked allelic-drive strategy described in this study can efficiently drive the preferred parasite refractory variant of the *FREP1* locus into a freely mating population of mosquitoes and render them robustly refractory to parasite infection, providing a promising foundation for future application in vector control.

### Looking forwards

In the current study, the linked allelic drive was inserted very near to the edited target site (126 bp) on the basis of practical considerations (that is, for creating equivalent donor and receiver congenic lines for optimal strain comparison). In general, however, the drive cassette could be deployed from various nearby locations relative to its targeted allelic cleavage site. For example, if the goal is to ensure the persistence of the allelic-drive system within a population, the gRNA-bearing cassette could be inserted (with or without a Cas9 transgene) within an exon of the target locus some distance away from the gRNA cut site along with function-restoring recoded sequences, into a neighbouring non-essential gene, or placed in closely linked intergenic regions. In addition, the allele-driving gRNA could be incorporated into gene cassettes designed for more localized effects. For example, although the linked drive tested here efficiently drove the Q224 edit to replace the chromosomally aligned *FREP1*^*GFP-L*^ allele (these two alleles share more than 1.2 kb of cassette homology on the intron side of the gRNA cut site), it was less efficient when combined with a WT allele that shares only 126 bp of intronic homology (as such a configuration eliminates a significant fraction of target alleles). Thus, when deployed in the latter configuration, the linked drive could act more locally to achieve a desired frequency of *FREP1*^*Q224*^ allele in a given population. Similarly, self-eliminating allelic-drive systems designed to impose even greater fitness costs could be used in cases when the goal is to completely eliminate the gene cassette from the population^54^. Thus, the driving gRNA could be readily incorporated into various drive architectures including full or split gene drives^47^, self-eliminating systems^54^, transcomplementing drives^55^ or integral drive^56,57^ configurations depending on specific objectives such as the size of the target population and how long one wished the drive to remain in the population.

In summary, we have shown that a single-nucleotide change in the *FREP1* gene is sufficient to confer a strong parasite-refractory phenotype and that inheritance of the protective *FREP1*^*Q224*^ allele can be efficiently biased using a linked allelic-drive system. A similar strategy could also be applied to primary African malaria vectors such as *A. gambiae* or could be used to convert insecticide-resistant alleles such as *knock-down-resistance* (*kdr*) mutation into the WT insecticide-sensitive allele^43,54^. In the future, such allelic drives might also be engineered to be self-eliminating by designing them to incur a fitness cost such that they act only transiently before disappearing from the population^54^, permitting localized allelic replacements with zero transgene end points.

## Methods

### Mosquito rearing and maintenance

The *A. stephensi* WT (UCISS2018) and transgenic *vasa*-Cas9 lines used in this study were shared by A. A. James’s laboratory (University of California Irvine)^58^. These two lines have been bred and stably maintained in the laboratory for over 30 generations. Mosquitoes were grown at 27 °C under standard conditions with 77% humidity and a 12-h day–night lighting cycle. Mosquito larvae were fed with a mixture of powdered fish food (TetraMin) and yeast (2:1; Red Star), and adults were supplied with 10% (wt/vol) sucrose. Five days after mating, females were fed with cold calf blood (Colorado Serum Company) using Hemotek blood-feeding facilities. Mosquitoes used for *P. falciparum* and *P. berghei* infections were reared in the Johns Hopkins Insectary Core facility from eggs to adults. Similar rearing conditions were used with additional cat food pellets being added to the regular larvae food mixture.

### Ethics statement

This study was carried out in strict accordance with the recommendations in the Guide to the Care and Use of Laboratory Animals of the US National Institutes of Health. The parasite challenge assay protocol was approved by the Animal Care and Use Committee of Johns Hopkins University (permit number #MO21H10). Commercial anonymous human blood from Interstate Blood Bank was used for parasite cultures and mosquito feeding, and informed consent was therefore not applicable. This protocol has been approved by the Johns Hopkins School of Public Health Ethics Committee. For mosquito rearing and blooding feeding, we followed procedures and protocols approved by the Institutional Biosafety Committee from the University of California San Diego, complying with all relevant ethical regulations for animal testing and research (protocol #S18147).

### Mosquito transgenesis

Microinjections were performed as previously described by injecting a mixture of donor plasmids into the pre-blastoderm *vasa*-Cas9 embryos^14^. Donor plasmids were injected at 250 ng μl^−1^ in the injection buffer (5 mM KCl and 0.1 mM sodium phosphate, pH 6.8) filtered by 0.22-μm filter. F_0_ females and males were separated into two cohorts when they pupated and outcrossed to WT UCISS2018 mosquitoes in pools. All F_1_ progeny were screened for whole-body RFP (*FREP1*^*RFP-Q*^ and *FREP1*^*RFP-gRNA-Q*^) or eGFP (*FREP1*^*GFP-L*^ or *FREP1*^*GFP-Q*^) under UV-fluorescence microscopy at late larval stages. Positive F_1_ transgenic mosquitoes were used for isolating single colonies by mating with WT counterparts. After egg laying, the transgenic founders were crushed with single-fly preparation buffer (49 μl lysis buffer + 1 μl proteinase K to a final concentration of 0.3 mg ml^−1^. The lysis buffer included 1 mM EDTA, 10 mM Tris, pH 8.2, and 25 mM NaCl) for genotyping.

The orthologous amino acid (L224) in the *A. stephensi* FREP1 (ASTEI02574) protein was identified by protein sequence alignment with its *A. gambiae* FREP1 (AGAP007031) orthologue, which corresponds to the L442 residue in the *A. gambiae* FREP1 protein^3^. To create a clean *FREP1*^*Q224*^ edit in *A. stephensi* and avoid mutagenesis caused by NHEJ repair, we inserted a gene cassette carrying a fluorescent marker (either *IE1-RFP-SV40* or *IE1-eGFP-SV40*) into the second intron of the *FREP1* locus with a separate plasmid expressing gRNA^Intron^ (5′-GCGACGACGATTGTAGACGCTGG-3′) targeting a site 126 bp upstream from the residue L224. The codon change from CTA (L224) to CAA (Q224) was included in the right homologous arm of the donor plasmid, downstream of the selection marker. With this arrangement, the *FREP1*^*Q224*^ edit only results from co-integration of the desired edit with the gene cassette when DSB end resection proceeds beyond this site (that is, further than 126 bp in the 3′ direction).

The linked allelic-drive cassette, *FREP1*^*RFP-gRNA-Q*^, has the same structure and integration site as the *FREP1*^*RFP-Q*^ cassette, but also includes a transgene encoding the allelic gRNA^L224^ (5′-GCTCCAGCTCGGTTAGCGTT-3′). This donor plasmid was injected into *A. stephensi vasa*-Cas9 pre-blastoderm embryos together with gRNA^Intron^ expressed from a separate plasmid, resulting in the *FREP1*^*RFP-gRNA-Q*^ gene cassette being inserted into the identical site in the *FREP1* second intron. F_1_ fluorescent-positive progeny were crossed with WT mosquitoes and collected for genotyping after egg laying using primers FREP1F333 and FREP1R334.

### Characterizing fitness costs

Fifty randomly selected adult mosquitoes were anaesthetized on ice to measure wing length, which was used as a surrogate examination for mosquito body size^13^. Mosquito fertility was calculated by crossing either transgenic females to the WT males to assess the female reproductivity, or crossing transgenic males to the WT females to examine the male contribution by single mating in plastic vials. At least 30 crosses were performed and the number of eggs produced from the single vial were counted and used for plotting. Three biological replicates were performed. Egg hatching rate was assessed by counting the number of young larvae from single mating at 6 days after egg laying. Significant differences were calculated by unpaired Student’s *t*-test. The lifespans of the transgenic mosquitoes were measured by collecting pupae, separating them into female and male cohorts, and setting up three cages for each cohort with 30 mosquito adults. Mosquitoes were supplied with a 10% sucrose solution. Dead mosquitoes were recorded and removed from the cages daily to calculate the survival rate until all mosquitoes had died. For cages measuring lifespans with mating and single-blood feeding, 30 randomly selected transgenic females were mated with 90 WT males for 5 days after emergence, then blood-fed with Hemotek, and dead females were continuously counted. Data were collected with Microsoft Excel 2019 (v16.30) and displayed by GraphPad Prism 8 (v8.2.1) with the ratio of survivability on each day. Statistical significance was calculated by Kaplan–Meier survivability analysis with pooled data from three biological replicates, with not significant (NS) *P* > 0.05, *0.01 < *P* < 0.05, **0.001 < *P* < 0.01, ***0.0001 < *P* < 0.001 and ****P < 0.0001. For assaying pupation time, approximately 100 randomly selected transgenic larvae were maintained in each tray and the number of pupae were recorded daily in three replicates. Adult emergence rates were also tabulated by enumerating female and male adults that emerged from the same tray.

### Parasite challenge assays

We determined the competency of *A. stephensi FREP1* congenic or control mosquitoes to serve as vectors for *P. falciparum* using artificial membrane feeding assays performed according to previously established methodology^13^. Transgenic or WT female mosquitoes were randomly selected and fed on NF54W *P. falciparum* gametocyte cultures at 37 °C by mixing with the fresh blood (red blood cells plus human serum; Interstate Blood Bank) to obtain a final gametocytaemia of 0.08% (low infection levels) or 0.15% (high infection levels). Seven-day-old adult mosquitoes were used for *P. falciparum* infections using a pumped water bath and glass membrane feeder with stretched parafilm for 1 h until mosquitoes were fully engorged. The adult mosquitoes used for parasite feeding were starved from a sugar source for 3–5 h in advance and any unfed or partially fed mosquitoes were removed immediately after blood feeding. At least three biological replicates were performed as independent replications, and each group contained at least 90 female mosquitoes, according to our established methodology^13^. Eight days after feeding with *P. falciparum* at 27 °C, the midguts were dissected in phosphate-buffered saline and stained with 0.2% mercurochrome to examine the number of oocysts developed in the midguts^13^. Fourteen days after *P. falciparum* blood feeding, pairs of salivary glands from individual females were dissected and put into the PCR tubes with 30 µl of phosphate-buffered saline followed by sporozoite counts according to a previously published protocol^13^. At least 60 successfully infected female mosquitoes were randomly selected, dissected and counted for oocysts in the midgut or sporozoites in the salivary glands. Two-tailed Mann–Whitney *U*-test was used to calculate *P* values for infection intensities, and Fisher’s exact test for infection prevalence. All parasite challenge assays were conducted with at least three independent biological replicates and the pooled numbers of individual replicates are presented, with the median number of oocysts and sporozoites shown in Figs. 3 and 4i, as the median provides the most definitive measure for distinguishing statistical differences in the distributions of parasite loads. GraphPad Prism 10.0 was used to present both infection intensities and prevalences.

All three *FREP1* transgenic mosquito lines and the controls were also fed on *P. berghei* (WT, ANKA clone 2.34)-infected 8-week old Swiss Webster mice at 19 °C to examine its infection potency to the rodent malaria parasite^13^. Female mosquitoes at 12–13 days after infection were collected to investigate oocyst loads in the midguts, and salivary glands were collected at 19–21 days post-infection to determine sporozoite loads.

### Assessment of drive efficiency

Allelic conversion rates were assessed separately through female and male lineages. To track the target chromosome, we combined the *vasa*-Cas9 with the *FREP1*^*GFP-L*^ allelic variant. The *vasa*-Cas9; *FREP1*^*GFP-L*^ mosquitoes were then outcrossed with *FREP1*^*RFP-gRNA-Q*^ transgenic mosquitoes to examine the germline allelic conversion rate. F_0_ crosses were set up with 25 females and 25 males (all randomly selected) in three replicates. F_1_ larvae were first screened for the presence of all fluorescence (*IE1-RFP*-marked *FREP1*^*RFP-gRNA-Q*^, *IE1-GFP*-marked *FREP1*^*GFP-L*^ and *3xP3-CFP*-marked *vasa*-Cas9) and then used for F_1_ crosses with WT *A. stephensi* in female and male cohorts with the crossing schema displayed in Extended Data Fig. 7. All F_2_ progeny were scored for the presence of each fluorescence. Progeny carrying only the *IE-GFP* receiver chromosome was subjected to genotyping by allele-specific PCR amplification (primers SeqF and SeqR) and Sanger sequencing, and SnapGene (v5.0.7) was used for Sanger sequencing analysis. Allele conversion efficiency was quantified with the percentage of GFP^+^RFP^−^ individuals carrying Q224 edit.

### Allelic competition cage experiments

An allele competition cage trial was conducted to test fitness cost among three *FREP1* allelic variants, including *FREP1*^*RFP-Q*^, *FREP1*^*GFP-Q*^ and *FREP1*^*GFP-L*^. Intercrosses were performed between pairs of these three transgenic lines to generate transheterozygous mosquitoes for seeding (1:1 allele frequency). Triplicate cages were seeded with 30 randomly selected mosquito couples consisting of age-matched (5 days) F_1_ transheterozygous adults, resulting in 50% frequency for each allele. Cages were blood fed 3 days after seeding to stimulate oviposition. Allele frequencies were calculated by counting the fluorescence marker or targeted NGS analysis at 10 consecutive generations. Half of the progeny were passed to the next generation for each generation, and the other half were used for allele frequency quantification.

### Multi-generational cage experiments

We set up non-overlapping cage trials to test the performance of the linked allelic-drive in combination with the congenic *IE1-GFP* intronic insertion allele using the cross scheme as illustrated in Fig. 4e to generate the F_1_ transheterozygous carrying *vasa*-Cas9, *FREP1*^*RFP-gRNA-Q*^ and *FREP1*^*GFP-L*^, with the latter being used as the receiver chromosome. Note that the donor and receiver chromosomes share over 1.2 kb of homology on the intronic side of the gRNA cut site, as well as continuous homology on the other side following the 224 codon. These transheterozygous were then seeded with homozygous *FREP1*^*GFP-L*^ at 1:1 ratio (randomly selected 25 transheterozygous females, 25 transheterozygous males, 25 *FREP1*^*GFP-L*^ females and 25 *FREP1*^*GFP-L*^ males), resulting in a 1:3 (*FREP1*^*RFP-gRNA-Q*^:*FREP1*^*GFP-L*^) allelic seeding ratio. To avoid mating bias, all adults were aged for 5 days before seeding and blood meals were offered 3 days after seeding. In each generation, 300 L_1_–L_2_ larvae (L_1_ denotes the first instar larvae) were randomly selected and reared to adults for establishing the next generation, another 300 L_1_–L_2_ larvae were selected for rearing to adulthood and next-generation deep sequencing analysis of the receiver allele (using a PCR amplification with the SeqF GFP-specific primer as indicated in Fig. 4a). Approximately 1,000 L_4_ larvae were also randomly selected for phenotyping by scoring the fluorescence marker (except for generation 1, some of the cages produced less than 1,000 eggs), and the rest of the larvae were counted for population size at L_4_. Total allelic frequencies as indicated in Fig. 4e,h were calculated by weighting the frequencies of receiver-specific allelic frequencies determined by NGS based on the proportions of individuals displaying donor (RFP^+^) versus receiver (GFP^+^) chromosome phenotypes.

As presented in Extended Data Fig. 9, multi-generational cage experiments were also performed using a WT receiver chromosome (Extended Data Fig. 9a), in which homozygous mosquitoes for the *FREP1*^*RFP-gRNA-Q*^ intronic cassette were crossed to a *vasa*-Cas9 strain carrying the WT L224 allele. Transheterozygous *vasa*-Cas9/+; *FREP1*^*RFP-gRNA-Q*^/WT-L224 mosquitoes were then used for seeding cages at the same allele frequency as that used in the congenic experiments shown in Fig. 4. Note that in this case, there was only 126 bp of homology between the donor and receiver chromosomes in contrast to the extended homology present for the congenic experiment shown in Fig. 4 (pink boxes in Extended Data Fig. 9b).

### Targeted NSG

Genomic DNA was extracted from 20 randomly selected mosquitoes with DNeasy Blood & Tissue Kits according to the manufacturer (Qiagen), followed by column purification and ready for PCR amplification. About 300 ng genomic DNA was used for PCR amplification with gene-specific primers, each containing deep sequencing adaptors at the 5′-terminal (Supplementary Table 5). The NGS DNA libraries were prepared with two rounds of PCR, and then subjected to 100-bp paired-end high-throughput sequencing with IGM (Institute of Genomic Medicine, University of California, San Diego) as we have previously published^59^. Raw reads were demultiplexed using the Barcode Splitter Script by IGM, then analysed with DSB classifiers that we have previously published using RStudio (v4.1.0)^59^.

### Reporting summary

Further information on research design is available in the Nature Portfolio Reporting Summary linked to this article.

## Online content

Any methods, additional references, Nature Portfolio reporting summaries, source data, extended data, supplementary information, acknowledgements, peer review information; details of author contributions and competing interests; and statements of data and code availability are available at 10.1038/s41586-025-09283-6.

## Supplementary information

Supplementary Information Supplementary Method, Supplementary Results, Supplementary Discussion, Supplementary Tables 1–11

Reporting Summary

## Source data

Source Data Fig. 2, 3, 4 and Source Data Extended Data Fig. 3, 4, 5, 6, 9

## Figures and Tables

**Extended Data Fig. 5:**
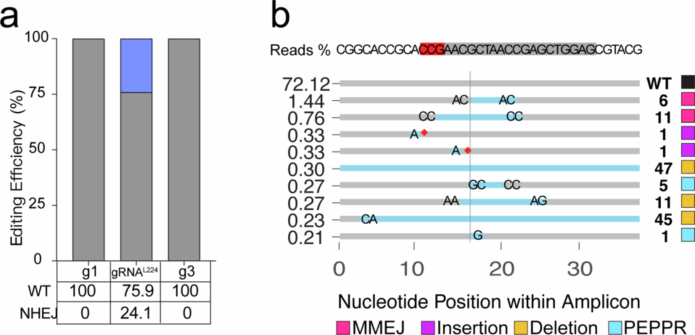
Screening for efficient allelic gRNAs. **a**, Editing efficiency of three potential candidate allelic gRNAs targeting the *FREP1* L224 site. Mosquito embryos were collected for microinjection with Cas9 protein and in vitro synthesized gRNA for microinjection. Two days old larvae hatched after microinjection were collected and pooled together for NGS to test gRNA cutting efficiencies. The graph shows the percentage of mutant indels enumerated below each bar. Gray bars represent WT alleles, and purple bars represent indels. **b**, Top mutant alleles generated with the gRNA^L224^. The plot shows a 38 bp reference sequence window centered by the expected gRNA cleavage site. The gray rectangle on the top row marks the protospacer, and PAM is marked with a red rectangle. The vertical gray line shows the expected DSB cutting site. Gray rectangles below the reference sequence are targeted genomic DNA *loci*, and blue rectangles show editing windows. Insertion alleles are marked with red diamonds. The left side displays the allelic read ratio (%), and the right shows the number of nucleotides that were deleted by color-coded allelic categories (see key below). Source data

## Data Availability

All plasmid sequences have been uploaded to the NCBI and are available online with the accession numbers: PP813873, PP813874, PP813875 and PP828956. NGS data have been deposited in the GenBank Sequence Read Archive database with the accession number PRJNA1112832. Source data have been provided for all raw data and model-fitting data generated in this study. NGS data were analysed by our previously published R program^59^. Source data are provided with this paper.

## Code availability

The code for mathematical modelling is available from the public data repository in GitHub (https://github.com/lambsUSP/FREP1).
