# Supplementary material for: Driving a protective allele of the mosquito FREP1 gene to combat malaria
Source: Nature. 2025 Jul 23;645(8081):746–54. doi: 10.1038/s41586-025-09283-6 (PMC12443604; doi:10.1038/s41586-025-09283-6)
Supplement: Supplementary file 1 — Supplementary Method, Supplementary Results, Supplementary Discussion, Supplementary Tables 1–11 [file 41586_2025_9283_MOESM1_ESM.docx]

**Supplementary Information**

**Model description**

The dynamics of the protective single-allelic variant of the mosquito *FREP1* gene were modeled considering discrete generations in agreement with the experiments. We considered a simplified framework in which the main genotype features studied and expected to occur in the experiment are denoted by five different letters, as indicated in Extended Data Table 6. We modeled the presence or absence of the *vasa*-Cas9 transgene using $A$ and $a$, respectively, where mosquitoes having genotypes $aa$, $Aa$ and $AA$ represent individuals lacking the transgene, heterozygous carriers, and homozygous carriers of *vasa*-Cas9, respectively. Additionally, we represent the *FREP1^RFP-gRNA-Q^, FREP1^GFP-Q^*, *FREP1^GFP-L^* and *FREP1^GFP-NHEJ^* alleles by the letters $B$, $C$, $D$ and $E$, respectively (Extended Data Table 7). In this framework, the genotype of each mosquito is represented by four different letters – e.g., $AaBD$ represents mosquitoes heterozygous for *vasa*-Cas9 and target chromosomes *FREP1^RFP-gRNA-Q^*/*FREP1^GFP-L^*. This simplified framework allows us to track phenotypic frequencies throughout non-overlapping multi-generational experiments, as shown in Source Data.

Cages were seeded with mosquitoes at a 1:1 phenotypic ratio, consisting of 50% male and female homozygous *FREP1^GFP-L^* mosquitoes (genotype $aaDD$), and 50% male and female offspring (genotype $AaBD$) derived from crosses between male mosquitoes homozygous *FREP1^RFP-gRNA-Q^* while lacking *vasa*-Cas9 (genotype $aaBB$) and female mosquitoes homozygous for *vasa*-Cas9 and *FREP1^GFP-L^* (genotype $AADD$).

We modeled the gene-drive performance by assuming that *FREP1^RFP-gRNA-Q^*/*FREP1^GFP-L^* mosquitoes carrying *vasa*-Cas9 (heterozygous $AaBD$ or homozygous $AABD$) might convert *FREP1^GFP-L^* into *FREP1^GFP-Q^* in the germline according to the rate $r.driving$, or into *FREP1^GFP-NHEJ^* according to the rate $r.NHEJ$. We assumed that homozygous *FREP1^GFP-NHEJ^* male and female mosquitoes are inviable and do not develop to the adult stage. Those remaining events in which the drive did not act at the *FREP1^GFP-L^* target site in the germline are accounted in the model according to the rate $r.neutral$ defined as $r.neutral$=1-$r.driving$-$r.NHEJ$. Additionally, based on the experiments reported in Fig. 2 (main text), we also assume no fitness cost associated with mosquitoes carrying different genotypes (aside from mosquitoes homozygous for *FREP1^GFP-NHEJ^*, as mentioned earlier).

At each experimental generation, we assumed that mosquitoes are well mixed and females mate only once. Because the *vasa*-Cas9 and *FREP1* loci are unlinked, we also assumed that the inheritance of alleles at these loci are independent (Extended Data Fig. 6). The genotypic frequency of each progeny, assumed to be composed of 50% males and 50% females, is obtained based on the genotypic frequency of the previous adult generation (Extended Data Tables 8, 9). Herein, we refer to the configuration described until here as the “standard model” (Extended Data Table 10).

We hypothesized that the drive has an additional influence on the dynamical system based on the data observed (Fig. 4d). A biologically plausible explanation to describe this observation could be a possible effect from *vasa-*Cas9 and the gRNA on *FREP1^GFP-L^* alleles during early embryo stages that would remain to the adult stages, as we discussed before in the main text. Thus, we considered a second model configuration named the “standard model + embryo effects” that adds the following effects to the experiment dynamics:

1. any embryo carrying a *FREP1^GFP-L^* allele which is exposed to *vasa-*Cas9 and gRNA (either from the father, the mother or the embryo itself, one from each) may have embryo effects (cleavage of *FREP1^GFP-L^* allele) with probability $p$;
2. in case of embryo effects, we have two distinct situations:
   1. if the embryo is either *FREP1^RFP-gRNA-Q^/FREP1^GFP-L^ or FREP1^GFP-Q^/FREP1^GFP-L^*, *FREP1^GFP-L^* allele may be converted into *FREP1^GFP-Q^* or *FREP1^GFP-NHEJ^* according to the previously defined rates, $r.driving$ or $r.NHEJ$, respectively; or,
   2. if the embryo is either *FREP1^GFP-L^/FREP1^GFP-L^* or *FREP1^GFP-NHEJ^/FREP1^GFP-L^*, since there is no "template allele" to accurately repair the *FREP1^GFP-L^* locus in response to cleavage, these individuals are assumed to be inviable.

Importantly, the absence of an allele to accurately repair the chromosome after cleavage was recently suggested by another modeling exercise describing gene-drive dynamics in non-overlapping generations^56^. In this application, because the targeted locus was located on the X chromosome (sex chromosome), the authors suggested that males and homozygous females carrying the targeted locus were inviable in the presence of the gene-drive elements since no allele could repair the cleave without avoiding new consecutive cuts, eventually generating NHEJ alleles. In the current study, this phenomenon is only possible due to embryo effects, since the genetic construct *FREP1^RFP-gRNA-Q^* encodes the Q224 locus together with the gRNA and, in the case of the *FREP1^GFP-L^* allele and *vasa*-Cas9, the target site may be converted to *FREP1^GFP-Q^*.

A third model configuration disregarded the paternal effects accounted for in the “standard model + embryo effects” configuration and is named “standard model + embryo effects - paternal effects” (Extended Data Table 10). Since all three model configurations are defined by three parameters, we compare them using their log-likelihood values after fitting them to data (Extended Data Table 10).

**Model fitting**

The model fitting was carried out considering the likelihood of observing, in each generation of each cage experiment, homozygous *FREP1^RFP-gRNA-Q^*, homozygous *FREP1^GFP^* and *FREP1^RFP-gRNA-Q^*/*FREP1^GFP^* mosquito counts, the L224, Q224 and NHEJ allele frequencies from 25 females and 25 males randomly selected for genotyping, and the number of mosquitoes carrying *vasa*-Cas9. To this end, we used Markov chain Monte Carlo (MCMC) methods to obtain posterior estimates and 95% credible intervals (CrIs) for each parameter (Extended Data Table 11). We assumed that the observed number of homozygous *FREP1^RFP-gRNA-Q^*, homozygous *FREP1^GFP^* and *FREP1^RFP-gRNA-Q^*/*FREP1^GFP^* mosquitoes, as well as the number of L224, Q224 and NHEJ alleles obtained after genotyping (Extended Data Table 9), can be modeled by multinomial distributions with probabilities given by their simulated frequencies at each generation and the number of screened mosquitoes (Extended Data Tables 8, 9). Additionally, we assumed that the observed number of mosquitos carrying *vasa*-Cas9 can be modeled by a binomial distribution with probabilities given by the simulated frequencies at each generation, given the number of screened mosquitoes (Extended Data Table 8). No prior distributions were used in the model fitting. All simulations were performed and analyzed in R.

**Stochastic simulations**

Simulated model trajectories were generated using a stochastic implementation of the discrete-generation model. At each generation, the offspring genotypic frequency was assumed to follow a multinomial distribution informed by the composite mated female genotype and the inheritance patterns of the $FREP1$ system. Because 300 larvae were sampled to seed the next generation, we assumed that 150 female and 150 male adults from each generation were randomly sampled to seed the next generation. Additional stochastic variations (following the same rationale) were integrated to the process to accommodate the number of screened mosquitoes at each generation (50 genotyped mosquitoes and number of screened mosquitoes according to the Extended Data Table 8).

**Results**

The model fitting indicates that the “standard model + embryo effects” has the highest log likelihood. Since all three models have the same number of parameters, this model is therefore selected to describe the cage experiment data (Extended Data Table 10). The posterior parameter values for this model are presented in Extended Data Table 11, and the multi-generational cage experiment and model simulation data is presented in Figs. 4g,h. Our results indicate that the conversion rate of *FREP1^GFP-L^* to *FREP1^GFP-Q^* in germline cells is 0.57 (0.53-0.62), in alignment with the lab experiments presented in Fig. 4d (main text), and that the rate of embryo effects is 0.83 (0.81-0.84). Additionally, our modeling framework suggests there is no fitness cost associated with mosquitoes carrying the *FREP1* constructs under laboratory conditions in this study.

**Discussion**

There is no direct experimental evidence on the level of maternal-effect homing associated with *vasa*-Cas9 in *An. stephensi*. When rates of homing or resistant allele generation resulting from maternally deposited Cas9 are inferred, this is based on maximizing the likelihood of the time-series marker phenotype data in generational cage experiments (i.e., the rates are inferred based on consistency with the observed data).

Notice that, the “embryo effect”, as defined, is not a direct measurable outcome. When it occurs, certain genotypes (*FREP1^GFP-L^*/*FREP1^GFP-L^* or *FREP1^GFP-NHEJ^*/*FREP1^GFP-L^*) are assumed to be inviable. If our assumptions are correct, these embryos therefore fail to develop into adults, or potentially not even evolve much after zygote formation, so the process remains hidden from direct observation.

Although the event is not directly observable, the “embryo effect” leaves a signature in the overall cage‑experiment dynamics. Without incorporating this mechanism, the model is not able to reproduce the genotype‑frequency trajectories seen in the laboratory data; including it in a relatively simple model enables an excellent fit, supporting the inferred values and assumptions.

**Supplementary Tabels**

**Supplementary Table 1. Oocysts intensity after *P. falciparum*** **infection at low infection level (0.08%)**

|  | **WT** | ***vasa*-Cas9** | ***FREP1^GFP-L^*** | ***FREP1^RFP-Q^*** | ***FREP1^GFP-Q^*** |
| --- | --- | --- | --- | --- | --- |
| *n* | 124 | 90 | 109 | 100 | 139 |
| *Mean* | 3.8 | 3.8 | 3.5 | 0.7 | 0.6 |
| *Median* | 3 | 3 | 3 | 0 | 0 |
| *Mann-Whitney U test (Two-tailed) p-values* | ns | ctl | ns | <0.0001 | <0.0001 |
| *Prevalence* | 86.3% | 81.1% | 79.8% | 28.0% | 31.8% |
| *Fisher’s exact p-values* | ns | ctl | ns | <0.0001 | <0.0001 |
| *Range* | 0-17 | 0-25 | 0-17 | 0-8 | 0-6 |
| *% Reduction in Median* | 0% | ctl | 0% | 100% | 100% |

**Supplementary Table 2. Sporozoites intensity after *P. falciparum*** **infection at low infection level (0.08%)**

|  | **WT** | ***vasa*-Cas9** | ***FREP1^GFP-L^*** | ***FREP1^RFP-Q^*** | ***FREP1^GFP-Q^*** |
| --- | --- | --- | --- | --- | --- |
| *n* | 40 | 40 | 40 | 40 | 40 |
| *Mean* | 4354 | 5339 | 4336 | 1035 | 950 |
| *Median* | 4080 | 4650 | 4050 | 0 | 0 |
| *Mann-Whitney U test (Two-tailed) p-values* | ns | ctl | ns | <0.0001 | <0.0001 |
| *Prevalence* | 87.5% | 82.5% | 77.5% | 35.0% | 35.0% |
| *Fisher’s exact p-values* | ns | ctl | ns | <0.0001 | <0.0001 |
| *Range* | 0-8600 | 0-15000 | 0-15000 | 0-8500 | 0-6000 |
| *% Reduction in Median* | 12.3% | ctl | 12.9% | 100% | 100.0% |

**Supplementary Table 3. Oocysts intensity after *P. falciparum*** **infection at high infection level (0.15%)**

|  | **WT** | ***vasa*-Cas9** | ***FREP1^GFP-L^*** | ***FREP1^GFP-Q^*** |
| --- | --- | --- | --- | --- |
| *n* | 52 | 37 | 42 | 43 |
| *Mean* | 30.4 | 33.2 | 30.3 | 8.5 |
| *Median* | 28 | 32 | 32 | 6 |
| *Mann-Whitney U test (Two-tailed) p-values* | ns | ctl | ns | <0.0001 |
| *Prevalence* | 100% | 100% | 97.6% | 86.0% |
| *Fisher’s exact p-values* | ns | ctl | ns | 0.007 |
| *Range* | 6-71 | 3-77 | 0-66 | 0-41 |
| *% Reduction in Median* | 12.5% | ctl | 1.6% | 81.3% |

**Supplementary Table 4. Sporozoites intensity after *P. falciparum* infection at high infection level (0.15%)**

|  | **WT** | ***vasa*-Cas9** | ***FREP1^GFP-L^*** | ***FREP1^GFP-Q^*** |
| --- | --- | --- | --- | --- |
| *n* | 39 | 39 | 39 | 39 |
| *Mean* | 38435.4 | 33978.5 | 30818.5 | 8566.2 |
| *Median* | 36120 | 33480 | 32640 | 5760 |
| *Mann-Whitney U test (Two-tailed) p-values* | ns | ctl | ns | <0.0001 |
| *Prevalence* | 100% | 94.9% | 100% | 87.2% |
| *Fisher’s exact p-values* | ns | ctl | ns | 0.047 |
| *Range* | 7740-91590 | 0-83160 | 3840-63360 | 0-39360 |
| *% Reduction in Median* | 0% | ctl | 2.5% | 82.8% |

**Supplementary Table 5. Primers used in this study.**

| **Name** | **Primer sequences (5' - 3')** |
| --- | --- |
| **FREP1^Q/L^-NGSF** | ACACTCTTTCCCTACACGACGCTCTTCCGATCTCCTTTCAGCTTGAGGCTGGC |
| **FREP1^Q/L^-NGSR** | GACTGGAGTTCAGACGTGTGCTCTTCCGATCTCTCGATCTGGCTGCAGTCCC |
| **FREP1F333** | CGGACAGAGGCAAAGAACGAC |
| **FREP1R334** | CGCCGTGAAAGAGTGATGAAAGA |
| **SeqF** | CTTGTACAGCTCGTCCATGCCGAG |
| **SeqR** | CTGGCTGCAGTCCCGCGGCA |

**Supplementary Table 6. Simplified model framework.**

| **Locus** | **Allele** | **Description** |
| --- | --- | --- |
| *vasa*-Cas9 | $A$ | Allele with *vasa*-Cas9 |
|  | $a$ | Allele without *vasa*-Cas9 |
| *FREP1* | $B$ | *FREP1^RFP-gRNA-Q^* allele |
|  | $C$ | *FREP1^GFP-Q^* allele |
|  | $D$ | *FREP1^GFP-L^* allele |
|  | $E$ | *FREP1^GFP-NHEJ^* allele |

**Supplementary Table 7. Phenotype of mosquitoes according to the simplified model framework.**

| **Genotype** | **Phenotype** |
| --- | --- |
| *BC, BD, BE* | *RFP+GFP* |
| *BB* | *RFP* only |
| *CC, CD, CE, DD, DE, EE* | *GFP* only |

**Supplementary Table 8. Number of screened mosquitoes in the multi-generational cage trials.**

| **Replicates** | **R1** | **R2** | **R3** |
| --- | --- | --- | --- |
| F_0_ | 100 | 100 | 100 |
| F_1_ | 148 | 262 | 1406 |
| F_2_ | 1253 | 1538 | 1061 |
| F_3_ | 1066 | 1221 | 1118 |
| F_4_ | 1036 | 1052 | 1434 |
| F_5_ | 1033 | 1178 | 1080 |
| F_6_ | 1427 | 1333 | 1027 |
| F_7_ | 1000 | 1053 | 1032 |
| F_8_ | 1065 | 1137 | 1068 |
| F_9_ | 1175 | 1309 | 1212 |
| F_10_ | 1158 | 1173 | 1230 |

**Supplementary Table 9. Calculated allele frequency in the multi-generational cage trials.**

| **Alleles** | **Q224** | | | **L224** | | | **NHEJ** | | |
| --- | --- | --- | --- | --- | --- | --- | --- | --- | --- |
| **Replicates** | **R1** | **R2** | **R3** | **R1** | **R2** | **R3** | **R1** | **R2** | **R3** |
| F_0_ | 25.00 | 25.00 | 25.00 | 75.00 | 75.00 | 75.00 | 0 | 0 | 0 |
| F_1_ | 74.76 | 74.82 | 71.80 | 23.21 | 15.90 | 23.30 | 2.03 | 9.28 | 4.90 |
| F_2_ | 88.90 | 66.40 | 77.53 | 8.93 | 28.94 | 17.84 | 2.98 | 4.66 | 4.64 |
| F_3_ | 84.28 | 76.73 | 76.49 | 12.18 | 15.59 | 16.28 | 3.54 | 7.68 | 7.23 |
| F_4_ | 87.31 | 90.39 | 90.19 | 11.03 | 7.13 | 7.28 | 1.67 | 2.48 | 2.53 |
| F_5_ | 88.38 | 93.33 | 84.37 | 10.94 | 6.07 | 11.41 | 0.68 | 0.60 | 4.22 |
| F_6_ | 89.11 | 94.36 | 89.36 | 9.73 | 4.59 | 8.96 | 1.16 | 1.05 | 1.68 |
| F_7_ | 94.85 | 90.85 | 94.58 | 3.08 | 7.04 | 3.68 | 2.07 | 2.11 | 1.74 |
| F_8_ | 91.81 | 92.79 | 98.82 | 7.85 | 6.62 | 0.68 | 0.34 | 0.59 | 0.50 |
| F_9_ | 87.42 | 94.52 | 96.31 | 8.91 | 3.20 | 1.34 | 3.67 | 2.28 | 2.35 |
| F_10_ | 87.26 | 98.76 | 97.87 | 12.20 | 0.70 | 1.63 | 0.54 | 0.54 | 0.50 |

**Supplementary Table 10. Model selection.**

| **Model** | **Loglikelihood** |
| --- | --- |
| standard model | -4219.358 |
| standard model + embryo effects | -1659.834 |
| standard model + embryo effects - paternal effects | -2046.054 |

**Supplementary Table 11. Model parameters, descriptions, and estimated posterior values and credible intervals for the best model.**

| **Variable** | **Description** | **Posterior [mean (95% CrI)]** |
| --- | --- | --- |
| *r. driving* | Conversion rate of *FREP1^GFP-L^* in *FREP1^GFP-Q^* in germline cells | 0.57 (0.53—0.62) |
| *r. NHEJ* | Conversion rate of *FREP1^GFP-L^* in *FREP1^NHEJ^* in germline cells | 0.06 (0.05—0.09) |
| *P* | Probability of embryo effects | 0.83 (0.81—0.84) |
