# Supplementary material for: Driving a protective allele of the mosquito FREP1 gene to combat malaria
Source: Nature. 2025 Jul 23;645(8081):746–54. doi: 10.1038/s41586-025-09283-6 (PMC12443604; doi:10.1038/s41586-025-09283-6)
Supplement: Supplementary file 2 — Reporting Summary [file 41586_2025_9283_MOESM2_ESM.pdf]

nature portfolio

Corresponding author(s): George Dimopoulos, Ethan Bier

Last updated by author(s): 05/20/2025

Reporting Summary

Nature Portfolio wishes to improve the reproducibility of the work that we publish. This form provides structure for consistency and transparency in reporting. For further information on Nature Portfolio policies, see our Editorial Policies and the Editorial Policy Checklist.

Statistics

For all statistical analyses, confirm that the following items are present in the figure legend, table legend, main text, or Methods section.

n/a	Confirmed	
☐	☒	The exact sample size (*n*) for each experimental group/condition, given as a discrete number and unit of measurement
☐	☒	A statement on whether measurements were taken from distinct samples or whether the same sample was measured repeatedly
☐	☒	The statistical test(s) used AND whether they are one- or two-sided<br>*Only common tests should be described solely by name; describe more complex techniques in the Methods section.*
☒	☐	A description of all covariates tested
☒	☐	A description of any assumptions or corrections, such as tests of normality and adjustment for multiple comparisons
☐	☒	A full description of the statistical parameters including central tendency (e.g. means) or other basic estimates (e.g. regression coefficient) AND variation (e.g. standard deviation) or associated estimates of uncertainty (e.g. confidence intervals)
☐	☒	For null hypothesis testing, the test statistic (e.g. *F*, *t*, *r*) with confidence intervals, effect sizes, degrees of freedom and *P* value noted<br>*Give P values as exact values whenever suitable.*
☐	☒	For Bayesian analysis, information on the choice of priors and Markov chain Monte Carlo settings
☒	☐	For hierarchical and complex designs, identification of the appropriate level for tests and full reporting of outcomes
☒	☐	Estimates of effect sizes (e.g. Cohen's *d*, Pearson's *r*), indicating how they were calculated

Our web collection on statistics for biologists contains articles on many of the points above.

Software and code

Policy information about availability of computer code

Data collection	Zeiss Stemi 2000 fluorescence microscope were used to acquire mosquito fluorescent images. Microsoft Excel 2019 (v16.30) was used for data collection.
Data analysis	Fiji (OS version) and Photoshop (Photoshop CC v20.0.7) were used to adjust contrast and brightness of images. GraphPad Prism 8 (v8.2.1) was used for data analysis and display. SnapGene (v5.0.7) was used for Sanger sequencing analysis. R studio (v4.1.0) was used for NGS data analysis. Mathematical modeling was conducted with Rstudio (V4.4.1) and the code for mathematical modeling is available from the public Data Repository in GitHub https://github.com/lambsUSP/FREP1.

For manuscripts utilizing custom algorithms or software that are central to the research but not yet described in published literature, software must be made available to editors and reviewers. We strongly encourage code deposition in a community repository (e.g. GitHub). See the Nature Portfolio guidelines for submitting code & software for further information.

Data

Policy information about availability of data

All manuscripts must include a data availability statement. This statement should provide the following information, where applicable:

- Accession codes, unique identifiers, or web links for publicly available datasets
- A description of any restrictions on data availability
- For clinical datasets or third party data, please ensure that the statement adheres to our policy

All plasmid sequences have been uploaded to NCBI and are available online with accession number: PP813873, PP813874, PP813875 and PP828956. Next-generation sequencing data have been deposited in the GenBank SRA database with accession number PRJNA1112832. Source data is provided for all raw data and model fitting data generated in this study. NGS data were analyzed by our previously published R program.

Research involving human participants, their data, or biological material

Policy information about studies with human participants or human data. See also policy information about sex, gender (identity/presentation), and sexual orientation and race, ethnicity and racism.

Reporting on sex and gender	This study has no experiment involves in human participants, their data or biological material.
Reporting on race, ethnicity, or other socially relevant groupings	Not applicable.
Population characteristics	Not applicable.
Recruitment	Not applicable.
Ethics oversight	Not applicable.

Note that full information on the approval of the study protocol must also be provided in the manuscript.

Field-specific reporting

Please select the one below that is the best fit for your research. If you are not sure, read the appropriate sections before making your selection.

☒ Life sciences ☐ Behavioural & social sciences ☐ Ecological, evolutionary & environmental sciences

For a reference copy of the document with all sections, see nature.com/documents/nr-reporting-summary-flat.pdf

Life sciences study design

All studies must disclose on these points even when the disclosure is negative.

Sample size	Our previous experience of similar analysis of HDR during cage trials suggested sequencing 20 individual mosquitoes is usually representative for bulk deep sequencing (Li et al., 2021, Li et al., 2024). We analyzed at least 40 individual fully engorged mosquitoes for parasite challenge assay (Dong et al., 2020).
Data exclusions	We excluded the single mating vials with no egg produced, since they might caused by unsuccessful mating. Regarding to the parasite challenge assay, we excluded all females without fully engorged for parasite infection analysis, since only the ones successfully fed with blood could be infected successfully.
Replication	For single mosquito Sanger sequencing, at least 25 individual mosquitoes were conducted, at least three biological replicates were performed. Three cages were run at the same time for cage trials. Other replication details are addressed in the text. All attempts at replication were successful.
Randomization	For each cross, at least 25 F2 progeny were randomly selected and subjected for Sanger sequencing to check the allelic copy efficiency. For cage trial populations, 25 females and 25 males were randomly collected from each cage at each generation.
Blinding	In all genetic experiments performed we had checked fluorescence presence in the bodies and eyes of mosquitoes. This type of scoring does not need the investigators to be blind as the evaluation of the phenotype is presence or absence of the fluorescence marker and there is no much room for interpretation that could be subjective.

Reporting for specific materials, systems and methods

We require information from authors about some types of materials, experimental systems and methods used in many studies. Here, indicate whether each material, system or method listed is relevant to your study. If you are not sure if a list item applies to your research, read the appropriate section before selecting a response.

Materials & experimental systems

n/a	Involved in the study
☒	☐ Antibodies
☒	☐ Eukaryotic cell lines
☒	☐ Palaeontology and archaeology
☐	☒ Animals and other organisms
☒	☐ Clinical data
☒	☐ Dual use research of concern
☒	☐ Plants

Methods

n/a	Involved in the study
☒	☐ ChIP-seq
☒	☐ Flow cytometry
☒	☐ MRI-based neuroimaging

Animals and other research organisms

Policy information about studies involving animals; ARRIVE guidelines recommended for reporting animal research, and Sex and Gender in Research

Laboratory animals	Transgenic mosquitoes being generated from vasa-Cas9 embryos at about 0.5 hours after egg laying, which was kindly shared by Dr. Anthony A. James's lab (University of California Irvine) and kept in our lab for more than 30 generations. Calf blood (Colorado Serum Co., Denver) and mice feeding was used for mosquitoes blood feeding.
Wild animals	No wild animals were used in this study.
Reporting on sex	Reporting on sex is not applicable for this study.
Field-collected samples	No field-collected samples were used in this study.
Ethics oversight	This study was carried out in strict accordance with the recommendations in the Guide to the Care and Use of Laboratory Animals of the National Institutes of Health. The parasite challenge assay protocol was approved by the Animal Care and Use Committee of Johns Hopkins University (permit number #MO21H10). Commercial anonymous human blood from Interstate Blood Bank was used for parasite cultures and mosquito feeding, and informed consent was therefore not applicable. This protocol has been approved by the Johns Hopkins School of Public Health Ethics Committee. For mosquito rearing and blooding feeding, we followed procedures and protocols approved by the Institutional Biosafety Committee from the University of California San Diego, complying with all relevant ethical regulations for animal testing and research (protocol #S18147).

Note that full information on the approval of the study protocol must also be provided in the manuscript.

Plants

Seed stocks	Not applicable.
Novel plant genotypes	Not applicable.
Authentication	Not applicable.
